# Mn‐Induced Support Stabilization and Ir Electronic Activation Enable Acid‐Stable, Low‐Loading IrO_2_ Water Oxidation

**DOI:** 10.1002/advs.75667

**Published:** 2026-05-11

**Authors:** Zhe Liu, Guoxin Ma, Shixiang Yu, Rui Jin, Fei Wang, Xinxin Wen, Mengxin Chen, Yani Ding, Jia Liu, Xinkai Guo, Diab Khalafallah, Hassan Fouad, Xiao Ren, Siwei Li

**Affiliations:** ^1^ State Key Laboratory of Fluorine & Nitrogen Chemicals School of Chemical Engineering and Technology Xi'an Jiaotong University Xi'an P.R. China; ^2^ Department of Physiology and Pathophysiology School of Basic Medical Sciences Health Science Center Xi'an Jiaotong University Xi'an P.R. China; ^3^ Institute of Electronic and Information Engineering of UESTC in Guangdong Dongguan P.R. China; ^4^ Beijing National Laboratory for Molecular Engineering College of Chemistry and Molecular Engineering Peking University Beijing P.R. China; ^5^ Institute of Sustainability for Chemicals, Energy and Environment (ISCE2) Agency for Science, Technology and Research (A*STAR) Jurong Island Singapore; ^6^ International Research Center for Renewable Energy State Key Laboratory of Multiphase Flow in Power Engineering School of Energy and Power Engineering Xi'an Jiaotong University Xi'an P.R. China; ^7^ Instrumental Analysis Center Xi'an Jiaotong University Xi'an P.R. China; ^8^ Mechanical Design and Materials Department Faculty of Energy Engineering Aswan University Aswan Egypt; ^9^ Health Sciences Department College of Applied Studies King Saud University Riyadh Saudi Arabia

**Keywords:** acidic oxygen evolution reaction, doping effect, low‐loading IrO_2_, Mn, support chemistry

## Abstract

The practical application of low‐iridium catalysts for the acidic oxygen evolution reaction (OER) is primarily constrained by the intertwined issues of inadequate activity and stability. Incorporation of Mn into such low‐iridium catalysts is effective, yet the underlying mechanism remains unclear. This study addresses the mechanistic role of Mn doping in enhancing the activity and stability of low‐loading IrO_2_/Co_3_O_4_ catalysts. By incorporating Mn^3+^ into the octahedral sites of Co_3_O_4_, Mn induces strong Mn─O covalency that reinforces the spinel lattice and stabilizes ultra‐low‐loading IrO_2_ nanoparticles, delivering a 51 mV reduction in overpotential and a six‐fold enhancement in operational stability compared to its undoped counterpart at a current density of 10 mA cm^−2^. In situ spectroscopic analyses and theoretical calculations decipher the dual role of Mn: it reinforces lattice integrity through strong covalent Mn─O bonds, suppressing ion leaching, while concurrently activating the Ir sites via interfacial Mn─O─Ir electron transfer, which optimizes intermediate adsorption and promotes the efficient oxide‐path mechanism (OPM). This work demonstrates that the targeted dual‐regulation of support chemistry establishes a general principle for designing high‐performance, low‐loading IrO_2_ catalysts for acidic OER.

## Graphical abstract

This study introduces a dual‐regulation strategy utilizing a Mn‐doped Co_3_O_4_ support to enhance the activity and stability of low‐loading IrO_2_ catalysts for acidic water oxidation. Mn induces strong Mn─O covalency to reinforce the spinel lattice and activates Ir sites via interfacial electron transfer. This approach provides a general principle for designing high‐performance, acid‐stable electrocatalysts.

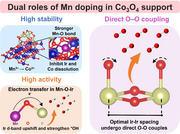

## 1 Introduction

The electrochemical oxygen evolution reaction (OER) under acidic conditions is a critical process for proton exchange membrane (PEM) water electrolysis, a promising technology for sustainable hydrogen production [1, 2, 3, 4, 5, 6]. However, the harsh acidic environment and sluggish OER kinetics impose a dual challenge: electrocatalysts must deliver both high intrinsic activity and long‐term structural stability to withstand corrosive conditions [7, 8, 9, 10, 11, 12]. Ir‐based materials currently set the benchmark catalysts for acidic OER, but suffer from their scarcity, high cost, and limited long‐term durability [7, 13, 14, 15]. Consequently, strategies that minimize Ir loading while enhancing performance via synergistic interactions with non‐noble supports have gained prominence [16, 17, 18, 19, 20, 21]. However, in the study of supported catalysts, research attention has heavily prioritized the chemical state of IrO_2_, including its valence and coordination environment, allocating substantial characterization and computational resources to these aspects [22, 23, 24]. By comparison, the properties of the support and the underlying stabilization mechanisms have been far less explored. In fact, targeted investigation into support chemistry through rational design of the composition and properties of non‐noble metal oxide supports offers a promising route to achieve highly active and stable acidic OER electrocatalysts under low iridium loading. Nevertheless, systematic studies in this regard remain largely insufficient.

Manganese (Mn) has emerged as a uniquely advantageous element in acidic OER catalysis. Mn‐based oxides are not only among the most promising non‐precious catalysts themselves, but also serve as effective supports for IrO_2_ and RuO_2_, significantly enhancing their catalytic performance [25, 26, 27, 28, 29]. Moreover, even if a small amount of Mn is employed as a dopant in support, the OER activity and stability in acidic media can be improved significantly [30, 31, 32, 33]. For instance, Mn‐doped Co_3_O_4_ spinel structures have demonstrated improved corrosion resistance and electronic conductivity compared to pristine Co_3_O_4_, enabling better dispersion and stabilization of Ir species [34]. Besides, our previous study showed that Mn dopant in Co_3_O_4_ altered the electronic structure and reduced the leaching of RuO_2_, leading to better activity and long‐term stability [24]. Despite these empirical advances, the atomic‐scale mechanisms underlying Mn's dual structural and electronic roles remain unresolved.

In this work, we present a case study in advanced support chemistry by introducing Mn into the octahedral sites of a Co_3_O_4_ support for IrO_2_/Co_3_O_4_ catalysts under acidic OER conditions. The resulting IrO_2_/Mn‐Co_3_O_4_ catalyst achieves a lower overpotential of 269 mV at a current density of 10 mA cm^−2^, which is 51 mV lower than that of the undoped counterpart, and demonstrates more than six‐fold operational stability. Through a combination of spectroscopic and theoretical methods, we elucidate a mechanism of Mn‐Induced Dual‐Regulation of Support Chemistry: Mn not only reinforces the structural integrity of the support via strong covalent Mn─O bonds, suppressing the leaching of Co and Ir, but also enables interfacial electron transfer through Mn─O─Ir bridges, which raises the Ir d‐band center and enhances the adsorption of *OH intermediates. This dual modulation facilitates the oxide‐path mechanism and promotes the formation of O─O bonds. Our findings reveal how atomic‐level doping can strategically tailor support properties to achieve highly active and stable low‐iridium acidic OER electrocatalysts.

## 2 Results & Discussions

### 2.1 Engineering Support Chemistry: Atomic Incorporation of Mn^3+^ and IrO_2_ Stabilization

**FIGURE 1:**
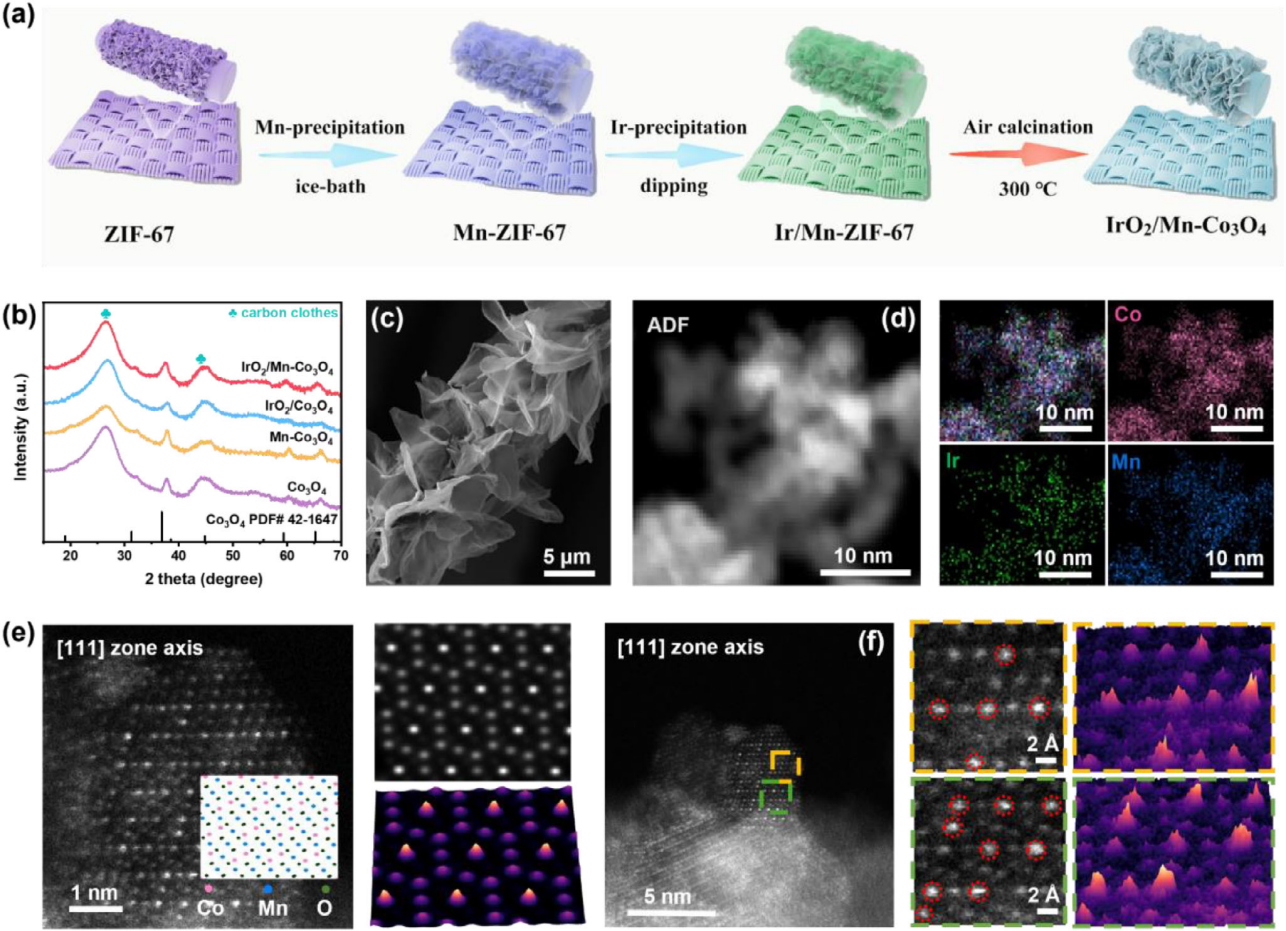
Synthesis and characterization of the catalysts. (a) Schematic of the fabrication process for IrO_2_/Mn‐Co_3_O_4_. (b) XRD patterns of the samples. (c) SEM images of IrO_2_/Mn‐Co_3_O_4_. (d) AC‐HAADF‐STEM and EDS mapping of IrO_2_/Mn‐Co_3_O_4_. (e) HAADF‐STEM of IrO_2_/Mn‐Co_3_O_4_ image taken along the [111] zone axis, accompanied by the corresponding structural model diagram and 3D surface electron density map. (f) HAADF‐STEM image of IrO_2_/Mn‐Co_3_O_4_, where the Ir atom is highlighted within the dotted red circle in the yellow and green square areas, followed by a 3D surface electron density map of the selected yellow and green square areas. Representative images are shown from at least three independent observations (n = 3).

To explore the influence of Mn doping on the spinel support, IrO_2_/Mn‐Co_3_O_4_ supported on carbon cloth (CC) was synthesized using ZIF‐67 as the precursor. The synthesis process involved sequential immersion in KMnO_4_ and IrCl_3_ aqueous solutions to incorporate Mn and Ir elements, followed by calcination at 300 °C in a muffle furnace (Figure 1a). Inductively coupled plasma optical emission spectrometry analysis (ICP‐OES) reveals that the contents of Ir and Mn were 14.5% and 2.9% (Table S1), respectively. For comparison, the control sample of IrO_2_/Co_3_O_4_ with similar Ir content (15.2%) was prepared under analogous conditions without KMnO_4_.

Besides for the peaks for CC, the X‐ray diffraction (XRD, Figure 1b) patterns of all samples primarily exhibit diffraction peaks consistent with the spinel Co_3_O_4_ phase (PDF# 42–1467), while the patterns for IrO_2_ is absent due to the small size of the Ir species. Scanning electron microscopy (SEM) shows IrO_2_/Mn‐Co_3_O_4_ and IrO_2_/Co_3_O_4_ generally inherit the nanosheet morphology of ZIF‐67, indicating that the synthetic steps for introducing Mn or Ir do not affect the morphology (Figure 1c and Figures S1–S3).

The fine structure of IrO_2_/Mn‐Co_3_O_4_ is first investigated by using aberration‐corrected high‐angle annular dark‐field scanning transmission electron microscopy (AC‐HAADF‐STEM) and corresponding high‐resolution energy‐dispersive spectroscopy (EDS) mapping (Figure 1d), which indicates that Ir, Co, and Mn disperse homogeneously at the scale of 10 nm. Upon closer observation, the HAADF‐STEM image taken along the [111] zone axis clearly reveals that the Mn‐Co_3_O_4_ possesses a highly ordered crystalline lattice (Figure 1e). The corresponding structural model diagram demonstrates the orderly arrangement of Co, Mn, and O atoms, represented by pink, blue, and green dots, respectively. Mn atoms clearly substitute partial Co sites within the lattice, confirming successful incorporation of Mn into the Co_3_O_4_ spinel structure. 3D surface electron density maps, derived from the HAADF‐STEM images, visualize atomic distribution, where the bright peaks indicate positions of atoms with heights proportional to atomic number or density. The obtained lattice image matches closely with the theoretical simulation of Mn‐doped spinel (Mn‐Co_3_O_4_), providing strong evidence for uniform Mn substitution into Co lattice sites.

Further magnified HAADF‐STEM images distinctly illustrate the structure of Ir species in IrO_2_/Mn‐Co_3_O_4_ (Figure 1f). Localized bright spots highlighted by red circles, accompanied by corresponding 3D electron density maps, indicate enhanced Z‐contrast regions consistent with ultra‐small IrO_2_ clusters (<2 nm) and several Ir single atoms anchored on the Mn‐Co_3_O_4_ support. These bright spots manifest as sharp peaks distributed uniformly, confirming that Ir exists predominantly as a single atom or extremely small cluster rather than aggregated nanoparticles, which is also supported by the HAADF‐STEM images taken in a different area (Figure S4). Moreover, HRTEM and corresponding EDS mapping of IrO_2_/Co_3_O_4_ (Figure S5) indicate that the IrO_2_ in the control group also exists as small clusters. The similar fine structure of IrO_2_/Mn‐Co_3_O_4_ and IrO_2_/Co_3_O_4_ lays the foundation for understanding the effect of Mn.

**FIGURE 2:**
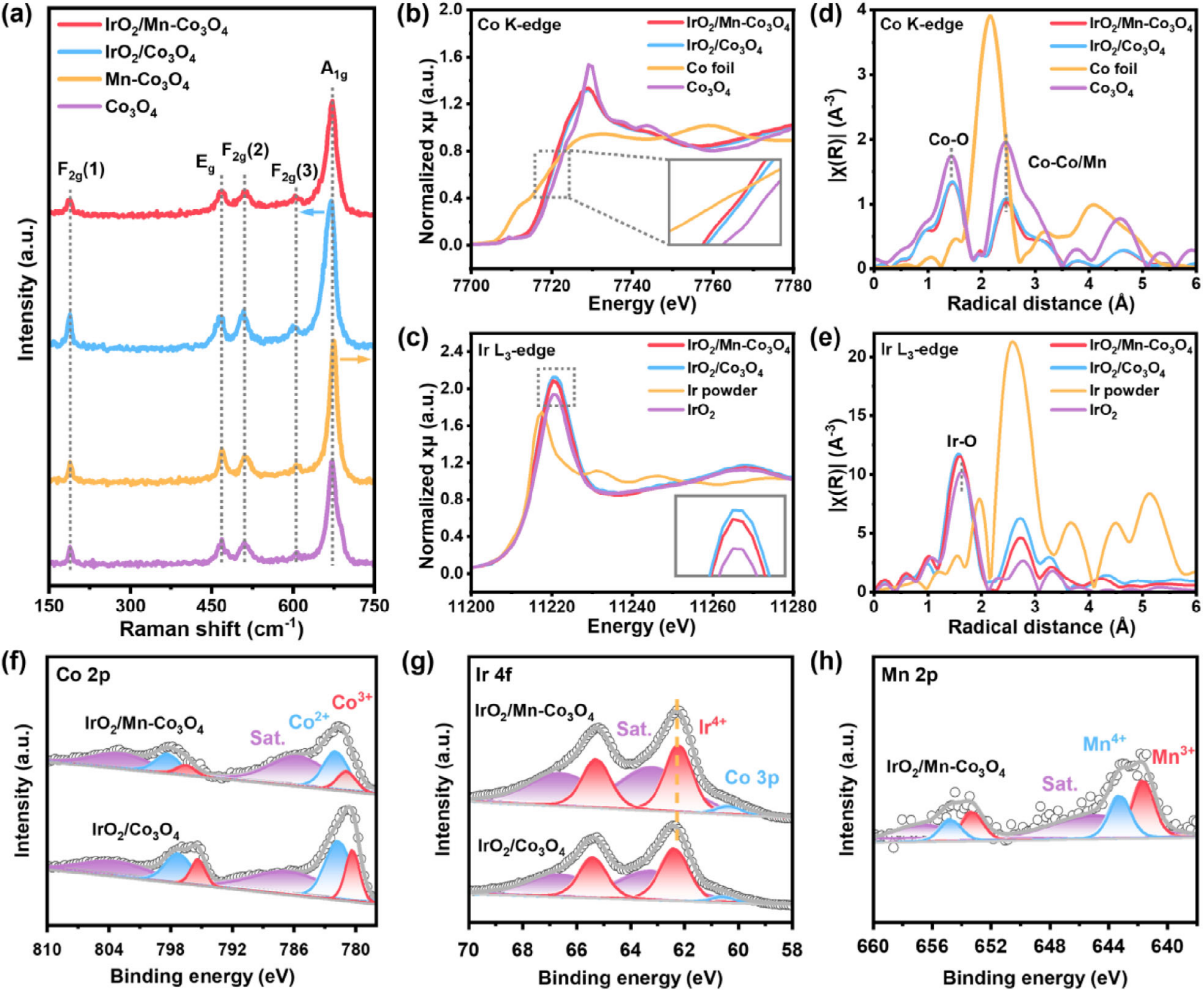
(a) Raman spectroscopy measurements for IrO_2_/Mn‐Co_3_O_4_, IrO_2_/Co_3_O_4_, Mn‐Co_3_O_4,_ and Co_3_O_4_. (b) Co K‐edge XANES of IrO_2_/Mn‐Co_3_O_4_ and IrO_2_/Co_3_O_4_ with other reference samples. (c) Ir L_3_‐edge XANES of IrO_2_/Mn‐Co_3_O_4_ and IrO_2_/Co_3_O_4_ with other reference samples. (d) Fourier transformed magnitudes of Co K‐edge EXAFS spectra for IrO_2_/Mn‐Co_3_O_4_ and IrO_2_/Co_3_O_4_ with other reference samples. (e) Fourier transformed magnitudes of Ir L_3_‐edge EXAFS spectra for IrO_2_/Mn‐Co_3_O_4_ and IrO_2_/Co_3_O_4_ with other reference samples. The inset zooms in on the white line feature. IrO_2_/Mn‐Co_3_O_4_ and IrO_2_/Co_3_O_4_ for XPS spectra: (f) Co 2p (g) Ir 4f (h) Mn 2p. Representative spectra are shown from three independent samples (n = 3).

Raman spectroscopy further elucidates the atomic‐scale structural perturbations induced by Mn and IrO_2_. In the Raman spectra (Figure 2a), the peaks observed at 188, 468, 519, 603, and 670 cm^−1^ are assigned to the F_2g_(1), E_g_, F_2g_(2), F_2g_(3), and A_1g_ vibration modes of pristine Co_3_O_4_ [35, 36, 37]. Upon Mn^3+^ doping, Jahn‐Teller distortion enhances the Co─O bond in the octahedral sites, blue‐shifting the A_1g_ mode, whereas the F_2g_(1) peak, dominated by tetrahedral sites vibrations, remains unchanged [38, 39]. This selective shift indicates that Mn^3+^ selectively substitutes for Co^3+^ (octahedral sites) in the octahedral sites rather than for Co^2+^ (tetrahedral sites) in the tetrahedral ones. Conversely, IrO_2_ decoration introduces surface tensile stress, elongating Co─O bonds and reducing vibrational frequencies, thereby shifting the A_1g_ peak toward a red shift. The coexistence of Mn doping and IrO_2_ decoration creates a competitive modulation of the local bonding environment. When these opposing effects reach equilibrium, the net shift in Raman peak positions becomes negligible, as observed experimentally.

The electronic structure of Ir and Co in IrO_2_/Mn‐Co_3_O_4_ and IrO_2_/Co_3_O_4_ was analyzed through X‐ray absorption fine structure spectra (XAFS). The rising edge of the Co K‐edge X‐ray absorption near‐edge structure (XANES) spectra exhibits a slight negative energy shift of IrO_2_/Mn‐Co_3_O_4_ compared to IrO_2_/Co_3_O_4_ (Figure 2b), indicative of a lower average oxidation state of Co atoms after Mn doping. This phenomenon can be primarily attributed to the partial substitution of octahedral Co^3+^ by Mn^3+^ ion in the octahedral sites of spinel Co_3_O_4_, consistent with previous reports [33, 34]. The Ir L_3_‐edge absorption maxima of both IrO_2_/Mn‐Co_3_O_4_ and IrO_2_/Co_3_O_4_ shifted toward higher energy values, closely aligning with the characteristic position of IrO_2_ (Figure 2c). This energy shift confirms the predominant +4 oxidation state of Ir species in both catalysts [40]. Notably, the slightly reduced intensity of the L_3_‐edge white line of IrO_2_/Mn‐Co_3_O_4_ relative to IrO_2_/Co_3_O_4_ suggests a stronger electron transfer via interfacial Ir─O─Mn compared to Ir─O─Co [34].

To further elucidate the atomic coordination environments of Ir and Co, Fourier‐transformed k^3^‐weighted extended X‐ray absorption fine structure (FT‐EXAFS) analyses were conducted. The Co K‐edge FT‐EXAFS spectra (Figure 2d) of both IrO_2_/Mn‐Co_3_O_4_ and IrO_2_/Co_3_O_4_ reveals that Co─O and Co─Co/Mn scattering paths at 1.9 Å and 2.8 Å, respectively. Quantitative EXAFS fitting reveals that the Co─O coordination number (CN) in IrO_2_/Co_3_O_4_ is 5.3, with an average bond length of 1.93 Å. Upon Mn incorporation, the Co─O CN decreases to 4.9 (Table S2), while the bond length remains constant at 1.93 Å. Additionally, the Co─Co/Mn CN decreases from 8.4 to 7.3, implying that Mn doping does not significantly alter the bond length but decreases the coordination number, exposing more active sites and thereby enhancing the catalytic activity. At the Ir L_3‐_edge, FT‐EXAFS analysis revealed dominant Ir─O coordination at 1.5 Å for both IrO_2_/Mn‐Co_3_O_4_ and IrO_2_/Co_3_O_4_, closely resembling the characteristic IrO_2_ configuration (Figure 2e). Moreover, the Ir‐Ir coordination at ∼2.7 Å confirms that Ir mainly exists as IrO_2_ clusters, consistent with the previous HADDF‐STEM observation. The conclusion drawn by FT‐EXAFS can also be supported by the wavelet transform EXAFS (WT‐EXAFS) analysis in Figure S6.

X‐ray photoelectron spectroscopy (XPS) was employed to probe the chemical states and electronic interactions of all surface elements. The survey spectra of both samples indicate the purity in terms of elements (Figure S7). The Co 2p spectrum (Figure 2f) exhibits two pairs of peaks: the dominant peaks at 780.3 and 795.4 eV are assigned to Co^3^^+^, while the minor peaks at 781.9 and 797.2 eV correspond to Co^2^^+^ [41, 42]. After Mn incorporation, all Co 2p peaks shift toward lower binding energies, accompanied by a marked reduction in the Co^3^^+^/Co^2^^+^ ratio to 39.1% from 47.4%. This confirms a lower average Co oxidation state, in agreement with the XANES results. Furthermore, Mn 2p spectra (Figure 2h) further reveal the co‐existence of Mn^3^^+^ and Mn^4^^+^ species whose binding energy locates at 641.6 and 643.2 eV, respectively [32]. The higher electronegativity of Mn induces a strong electron‐withdrawing effect, redistributing electron density toward Mn─O bonds and weakening adjacent Co─O interactions, which is further confirmed by the negative shift of lattice O species in the O 2p spectrum (Figure S8). Finally, Ir 4f spectra (Figure 2g) of IrO_2_/Mn‐Co_3_O_4_ show characteristic peaks at 60.4 and 63.4 eV, corresponding to Ir^4+^ (4f 7/2 and 4f 5/2) [43]. Compared to IrO_2_/Co_3_O_4_, the peaks shift toward lower binding energies, indicating an increased electron density around the Ir atoms induced by Mn doping. The electron‐rich IrO_2_ may facilitate the adsorption and desorption of reactive intermediates during the catalytic cycle, ultimately enhancing the OER activity.

Furthermore, the electronic structure of IrO_2_/Mn‐Co_3_O_4_ and IrO_2_/Co_3_O_4_ is investigated theoretically by analyzing the charge density differences between IrO_2_ and Co_3_O_4_ in the two systems. It is evident that Mn doping enhances the electron transfer between IrO_2_ and the support (Figure S9). To provide a more specific and quantitative comparison, we calculated the Bader charges (Table S3). The results indicate that the electron count associated with Ir increases significantly after Mn doping, which aligns with the findings from XPS and XAS analyses. With the incorporation of Mn, the electron transfer from the support to the IrO_2_ is further enhanced via interfacial Ir‐O‐Mn. This leads to a reduction in the oxidation state of Ir, increasing its oxygenophilicity and consequently enhancing the adsorption of oxygen‐containing intermediates.

Based on HADDF‐STEM, Raman, XAFS, XPS, and DFT calculations, it can be concluded that the incorporation of Mn not only reduces the average state of Co in Co_3_O_4_ supports by substituting the octahedral Co^3+^, but also endows IrO_2_ clusters with a slightly higher valence state and electron‐deficient structure.

### 2.2 Dual‐Enhanced OER Performance of IrO_2_/Mn‐Co_3_O_4_ Through Modified Support Chemistry

**FIGURE 3:**
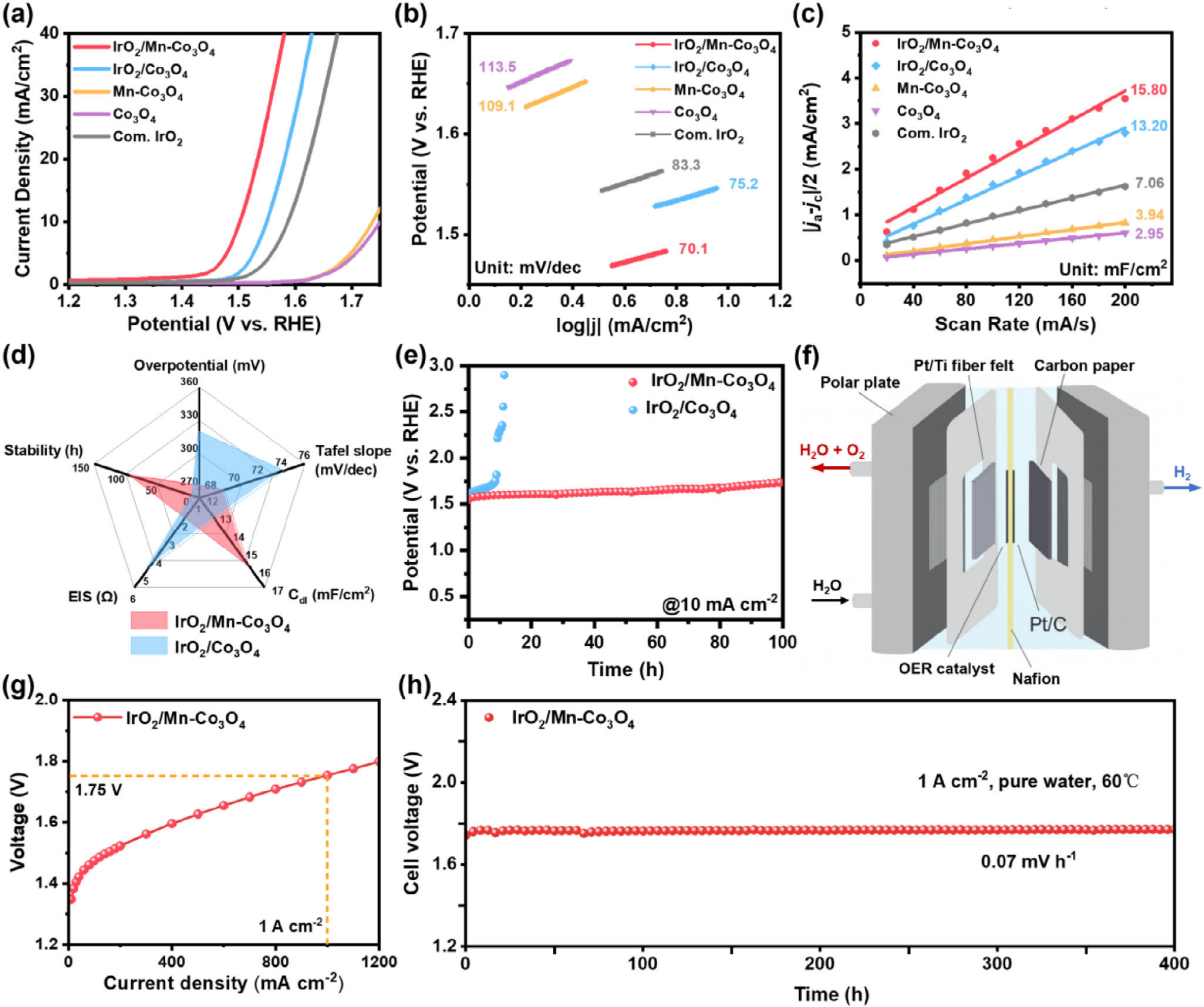
Electrocatalytic OER performance of the as‐made electrocatalysts in 0.5 m H_2_SO_4_. (a) LSV polarization curves (85% *iR*‐corrected) of the prepared samples. (b) Tafel plots for the corresponding samples. (c) Plots showing the extraction of the *C*_dl_. (d) Performance comparison of IrO_2_/Mn‐Co_3_O_4_ and IrO_2_/Co_3_O_4_. (e) The chronopotentiometry curves of IrO_2_/Mn‐Co_3_O_4_ and IrO_2_/Co_3_O_4_ at a current density of 10 mA/cm^2^. (f) Schematic of the PEMWE cell. (g) Polarization curves of the PEMWE device with IrO_2_/Mn‐Co_3_O_4_ (Ir loading: ∼0.5 mg_Ir_ cm^−2^) as anode catalysts at 60°C in ultrapure water. (h) Chronopotentiometry curves of the PEMWE device with IrO_2_/Mn‐Co_3_O_4_ as anode catalysts operated at 1000 mA cm^−2^ at 60°C in ultrapure water. Representative curves are shown from three independent experiments (n = 3).

The OER performance of IrO_2_/Mn‐Co_3_O_4_, IrO_2_/Co_3_O_4_, bare spinel oxides, and commercial IrO_2_ (Com. IrO_2_) was tested by using a typical three‐electrode system in 0.5 m H_2_SO_4_ electrolyte. Before testing, the reference electrode has been calibrated experimentally according to literature (Figure S10) [44]. Of note is that key synthesis parameters influencing OER performance, such as the KMnO_4_ concentration during Mn doping, were systematically optimized prior to comparative evaluation of the IrO_2_/Mn‐Co_3_O_4_ catalysts (Figure S11). As shown in the linear sweep voltammetry (LSV) curves in Figure 3a, the IrO_2_/Mn‐Co_3_O_4_ catalyst exhibits the best catalytic activity, achieving a low overpotential of only 269 mV at a current density of 10 mA/cm^2^. This performance significantly surpasses those of IrO_2_/Co_3_O_4_ (320 mV), Mn‐Co_3_O_4_ (459 mV), Co_3_O_4_ (521 mV), and Com. IrO_2_ (357 mV). Meanwhile, IrO_2_/Mn‐Co_3_O_4_ presents the lowest Tafel slope of 70.1 mV/dec among all catalysts, which is smaller than IrO_2_/Co_3_O_4_ (75.2 mV/dec), Mn‐Co_3_O_4_ (109.1 mV/dec), Co_3_O_4_ (113.5 mV/dec), and Com. IrO_2_ (83.3 mV/dec), suggesting that the simultaneous incorporation of Mn dopants and IrO_2_ nanoparticles significantly accelerates OER kinetics by enhancing charge‐transfer efficiency and optimizing active sites. (Figure 3b).

To further evaluate the charge transfer resistance (*R*_ct_), electrochemical impedance spectroscopy (EIS) is carried out under the same potential. Compared with IrO_2_/Co_3_O_4_ (4.94 Ω), Mn‐Co_3_O_4_ (66.88 Ω), Co_3_O_4_ (67.03 Ω), and commercial IrO_2_ (8.07 Ω), IrO_2_/Mn‐Co_3_O_4_ exhibits the lowest charge transfer resistance, which is only 2.12 Ω, highlighting the essential roles of Mn doping and IrO_2_ decoration in promoting electrical conductivity and facilitating electron transport. (Figures S12 and S13). To better understand the intrinsic activity enhancement, electrochemical double‐layer capacitance (*C*_dl_), correlated to the electrochemically active surface area (ECSA), was measured through cyclic voltammetry (CV) test (Figure S14). As depicted in Figure 3c, IrO_2_/Mn‐Co_3_O_4_ exhibits the highest *C*_dl_ of 15.80 mF/cm^2^, substantially larger than those of IrO_2_/Co_3_O_4_ (13.20 mF cm^−2^), Mn/Co_3_O_4_ (3.94 mF/cm^2^), Co_3_O_4_ (2.95 mF/cm^2^), and commercial IrO_2_ (7.06 mF/cm^2^). After ECSA normalization, IrO_2_/Mn‐Co_3_O_4_ retains superior intrinsic catalytic activity (Figure S15). Moreover, the mass activity normalized by Ir loading further verifies the superior catalytic efficiency of IrO_2_/Mn‐Co_3_O_4_ (106.1 A g_Ir_^−1^ at 1.53 V), higher than IrO_2_/Co_3_O_4_ (92.1 A g_Ir_^−1^).

It is essential to assess stability and long‐term durability in the practical application in water splitting [45, 46]. The overpotential of IrO_2_/Mn‐Co_3_O_4_ only reduces 30 mV at 10 mA·cm^−2^ after 1000 CV cycles (Figure S16). Notably, the long‐term durability is greatly improved from 15 h IrO_2_/Co_3_O_4_ to about 100 h of IrO_2_/Mn‐Co_3_O_4_, suggesting that a small amount of Mn in support can greatly affect the stability (Figure 3e). This is critically important for OER in acidic media. Quantitative ICP‐OES analysis of the electrolyte after the 100 h durability test reveals elemental retention rates of 55.8% for Co, 85.3% for Mn, and 82.7% for Ir (Table S4), further confirming the structural robustness of the Mn‐doped composite. To systematically evaluate the synergistic optimization mechanism of Mn doping on catalytic performance, Figure 3d comprehensively compares the electrochemical metrics of IrO_2_/Mn‐Co_3_O_4_ and IrO_2_/Co_3_O_4_. IrO_2_/Mn‐Co_3_O_4_ demonstrates superior performance across all critical parameters. This comparative analysis conclusively demonstrates the pronounced “dual‐functional” effect of Mn doping, which not only enhances the intrinsic activity of the catalyst but also effectively mitigates the corrosion of active species by acidic media, thereby reinforcing its long‐term stability under harsh acidic conditions.

Building upon the exceptional catalytic performance and stability of IrO_2_/Mn‐Co_3_O_4_ in three‐electrode systems, we further evaluated its practical viability by constructing a proton exchange membrane water electrolyzer (PEMWE). The PEMWE employed ultrapure water feed and Pt/C and IrO_2_/Mn‐Co_3_O_4_ as cathode/anode catalysts (Figure 3f), respectively. As shown in Figure 3g, the current‐voltage polarization curves reveal that the IrO_2_/Mn‐Co_3_O_4_‐based PEMWE achieves a cell voltage of 1.75 V at 1 A cm^−2^ under 60°C operating conditions. Notably, these PEMWE measurements were conducted as a proof‐of‐concept device evaluation, without device‐level optimization of Ir loading. Despite this, the system demonstrates remarkable operational stability, maintaining continuous operation for over 400 h at 1000 mA cm^−2^ (Figure 3h) with a minimal voltage degradation rate of approximately 0.07 mV h^−1^. Comparative analysis with previously reported PEMWE catalysts (Table S5) confirms that the IrO_2_/Mn‐Co_3_O_4_ composite exhibits superior overall performance metrics, combining both high efficiency and exceptional durability in practical electrolyzer applications.

### 2.3 Unraveling the Mn‐Mediated Stabilization Mechanism toward OER

**FIGURE 4:**
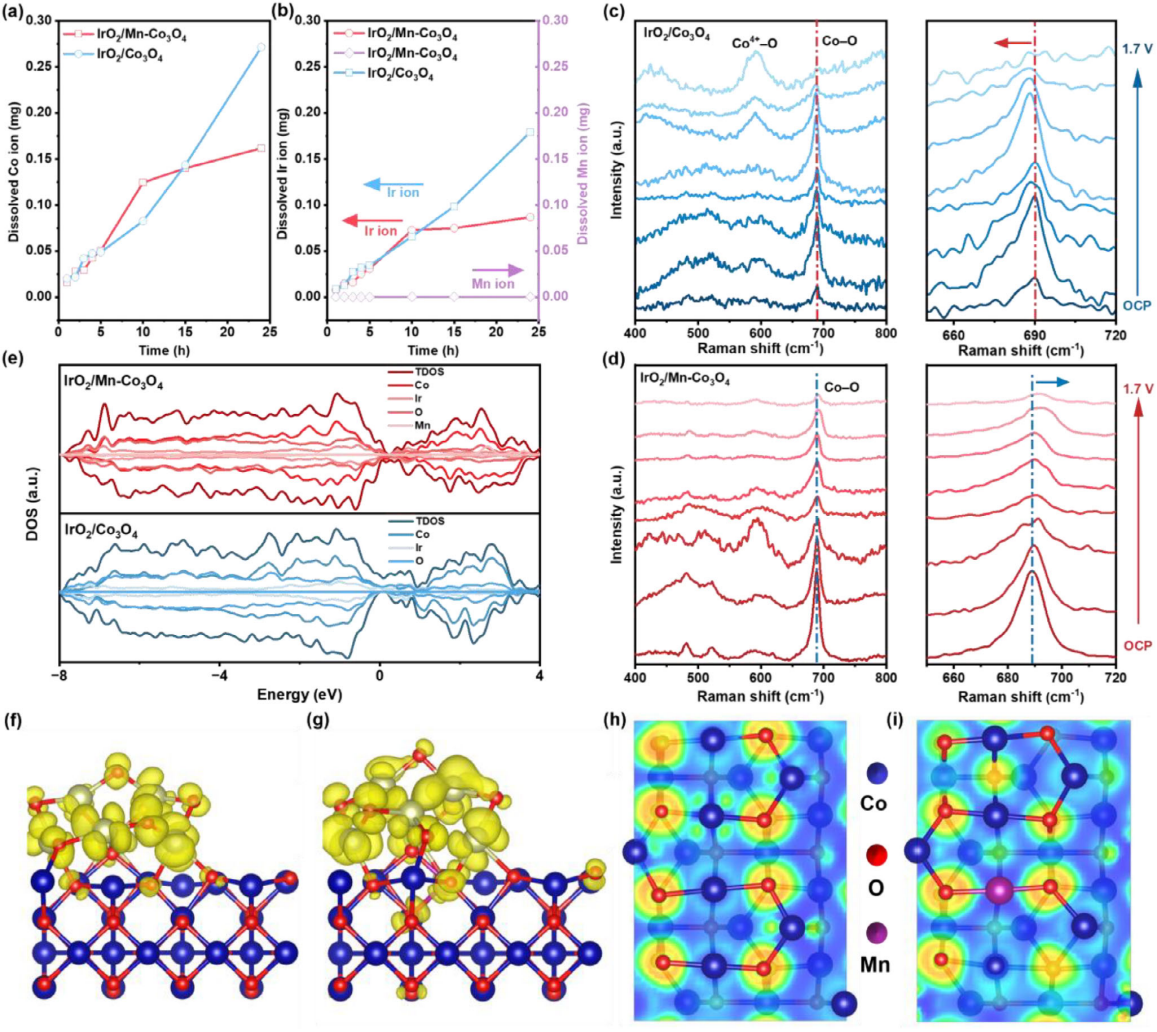
Dissolved ions concentrations measured for IrO_2_/Mn‐Co_3_O_4_ (0.750 mg cm^−2^) and IrO_2_/Co_3_O_4_ (0.725 mg cm^−2^) in 0.5 m H_2_SO_4_ electrolyte by ICP‐OES. (a) Co ions (b) Ir (left‐*y*‐axis) and Mn (right‐*y*‐axis). (c, d) in situ Raman spectroscopy measurements for IrO_2_/Mn‐Co_3_O_4_ and IrO_2_/Co_3_O_4_ recorded during acidic OER from 1.1 to 1.7 V versus RHE in 0.5 M H_2_SO_4_. (e) DOS plots of Co, Ir, O, and Mn states in IrO_2_/ Mn‐Co_3_O_4_ and DOS plots of Co, Ir, and O states in IrO_2_/Co_3_O_4_. Wave functions of the band‐edge states at the Γ point (isosurface level = 3e^−9^) (f) IrO_2_/Co_3_O_4_ and (g) IrO_2_/Mn‐Co_3_O_4_. 2D ELF contour plots for (h) IrO_2_/ Co_3_O_4_ and (i) IrO_2_/Mn‐Co_3_O_4_. The experimental data in (a‐d) represent results from three independent measurements (n = 3).

In order to further elucidate the mechanism by which Mn doping enhances the stability of the catalyst, we employed ICP‐OES to monitor the time‐evolution dissolution behavior of metal ions in solution during the acidic OER reaction for IrO_2_/Mn‐Co_3_O_4_ and IrO_2_/Co_3_O_4_ (Figure 4a,b). This suggests that Mn not only avoids causing lattice damage to the material but also functions as a structural stabilizer. In contrast, although Ir and Co in both samples dissolved fast in the first 10 h, after prolonged reaction times, the dissolved Co and Ir ions from IrO_2_/Mn‐Co_3_O_4_ were significantly lower than those from the undoped IrO_2_/Co_3_O_4_, which can also be supported by SEM and TEM of post‐reaction samples (Figures S17–S19). These results indicate that Mn doping effectively suppresses the dissolution of Co_3_O_4_ and IrO_2_, thereby enhancing the structural and chemical stability of the catalyst in an acidic electrolytic environment.

The in situ Raman spectroscopy data (Figure 4c,d) reveal distinct structural evolution trends for the IrO_2_/Co_3_O_4_ and IrO_2_/Mn‐Co_3_O_4_ catalysts under increasing applied potentials. For the undoped IrO_2_/Co_3_O_4_, the Co─O vibrational peak near 690 cm^−1^ exhibits a redshift (toward lower wavenumbers) with increasing potential. This redshift correlates with a weakening of Co─O bonds, likely driven by the oxidation of Co^3+^ to Co^4+^ under acidic OER conditions. Furthermore, the in situ Raman spectra revealed a progressive intensity enhancement of the Co─O vibrational mode at ∼590 cm^−1^ under increasing potentials, which is attributed to the formation of high‐valent Co^4+^ species during OER [47, 48, 49]. This observation aligns with the structural instability of undoped Co_3_O_4_, as the partial oxidation of Co^3+^ to Co^4+^ disrupts the native Co_3_O_4_ spinel framework (e.g., Co^3+^ [Co^2+^Co^3+^] O_4_ → Co^4+^O_2_‐like configurations). The intensified ∼590 cm^−1^ mode likely corresponds to symmetry‐breaking vibrations induced by Co^4+^‐O bond contraction and local lattice distortion, consistent with prior reports on metastable Co^4+^‐rich oxide phases in acidic OER [48, 50, 51]. The formation of higher‐valent Co^4+^ species elongates Co─O bonds due to reduced electron density and increased lattice oxygen participation in OER (e.g., *O → O_2_), accelerating structural degradation. The enlarged graph (660–720 cm^−1^ region) further confirms this trend, where the peak position progressively shifts leftward (∼4 cm^−1^ at OCP to 1.7 V), accompanied by intensity attenuation, indicative of bond rupture and oxygen vacancy generation.

In contrast, the Mn‐doped IrO_2_/Mn‐Co_3_O_4_ demonstrates a blueshift (toward higher wavenumbers) for the Co─O peak under identical conditions. The blueshift (∼4 cm^−^^1^ at OCP to 1.7 V) signifies strengthening of Co─O bonds, attributed to Mn^3+^ doping‐induced electronic modulation [33]. Mn species likely stabilize the Co^3+^/Co^4+^ redox couple through charge compensation, suppressing oxygen vacancy formation. Additionally, the stronger covalent Mn─O bonds (versus Co─O) mitigate lattice oxygen loss by stabilizing the Co_3_O_4_ framework [32]. The retained peak intensity in the 660–720 cm^−1^ region further supports enhanced structural integrity. The electrochemical stability improvement (100 h for Mn‐doped catalyst) aligns with these observations. The Mn‐induced Co─O bond strengthening and suppressed lattice oxygen participation collectively inhibit acid corrosion and phase transitions. The stability enhancement is further rationalized by the synergistic IrO_2_/Mn‐Co_3_O_4_ interactions, which optimize charge transfer kinetics and reduce overpotential‐driven structural stress.

Multiple theoretical calculations have been employed to deeply understand the doping effect of Mn on OER stability. Notably, DFT models were constructed using the as‐prepared lattice/cluster motif as an initial framework, while the mechanistic conclusions are constrained by post‐OER characterization and operando spectroscopic evidence of the stabilized active state. As can be seen from the DOS (Density of States) plot (Figure 4e), the electronic states of Co are generally higher than those of Ir across various energy ranges due to the significantly larger number of Co atoms compared to Ir atoms in the model. However, near the Fermi level, the electronic states of Co exhibit a noticeable decrease, even falling below those of Ir. This indicates that the reaction activity of Ir is significantly higher than that of Co, establishing Ir as the primary active site for the catalytic reaction, while Co functions as a cocatalyst and support. Mn primarily introduces new unoccupied states in the DOS above the Fermi level. The d‐band center values further corroborate this; the d‐band center of Mn lies relatively close to the Fermi level compared to the others, directly demonstrating that Mn introduces a portion of new density of states within the unoccupied orbitals. This increase in conduction band electronic states enhances the adsorption of oxygen‐containing intermediates by the support, thereby improving the stability of the system during the acidic OER process.

Furthermore, the CBM (Conduction Band Minimum) of IrO_2_/Co_3_O_4_ and IrO_2_/Mn‐Co_3_O_4_ was visualized (Figure 4f,g) to elucidate the effect of Mn doping on the electronic states of the catalyst. Clearly, compared to pure Co_3_O_4_, a higher concentration of unoccupied orbitals is localized around Mn atoms, consistent with the findings from the DOS plots and d‐band center analysis. This demonstrates that Mn possesses significantly stronger oxygen affinity within this system, which facilitates lattice oxygen regeneration and thereby maintains the structural stability. The calculated oxygen vacancy formation energies (E_v_) are presented in Figure S20. The E_v_ for the lattice oxygen adjacent to the Mn site increases significantly to 2.317 eV, compared to 1.110 eV for pristine Co_3_O_4_. This provides direct thermodynamic evidence that Mn doping effectively suppresses the leaching of lattice oxygen by increasing the energy barrier for vacancy formation. Concurrently, the enhanced oxygen affinity of Mn strengthens the Mn‐O‐Ir interaction, leading to a tighter coupling between the IrO_2_ and the support. Consequently, Ir receives a greater number of electrons from the support.

Conversely, in IrO_2_/Mn‐Co_3_O_4_, Mn (purple) substitutes for some Co positions, significantly enhancing electron localization around Mn. The ELF value between Mn and O is higher, and the electron cloud is more evenly distributed between Mn and O, unlike the Co─O bond, where electrons are primarily localized on the O atom. The Mn─O bond exhibits a stronger covalent character. Since covalent bonds are inherently more stable than ionic bonds, the enhanced covalency of Mn─O bonds contributes to greater overall lattice stability, reducing the likelihood of metal dissolution and structural collapse, leading to higher stability of IrO_2_/Mn‐Co_3_O_4_.

The effect of Mn doping on the OER stability can be summarized as below: after doping with Mn, which exhibits a stronger oxygen affinity, the structural stability of the spinel support can be enhanced through the formation of Mn‐O bonds with greater covalent character. Additionally, the interaction between IrO_2_ and the support is strengthened via the formation of Mn‐O‐Ir bonds at the interface. This effect further mitigates lattice expansion in the in situ OER catalyst, slows down the dissolution of Ir and Co species, and ultimately enhances the overall OER stability.

### 2.4 Electronic Modulation and Promotion of the Oxide‐Path Reaction through Support Modulation

**FIGURE 5:**
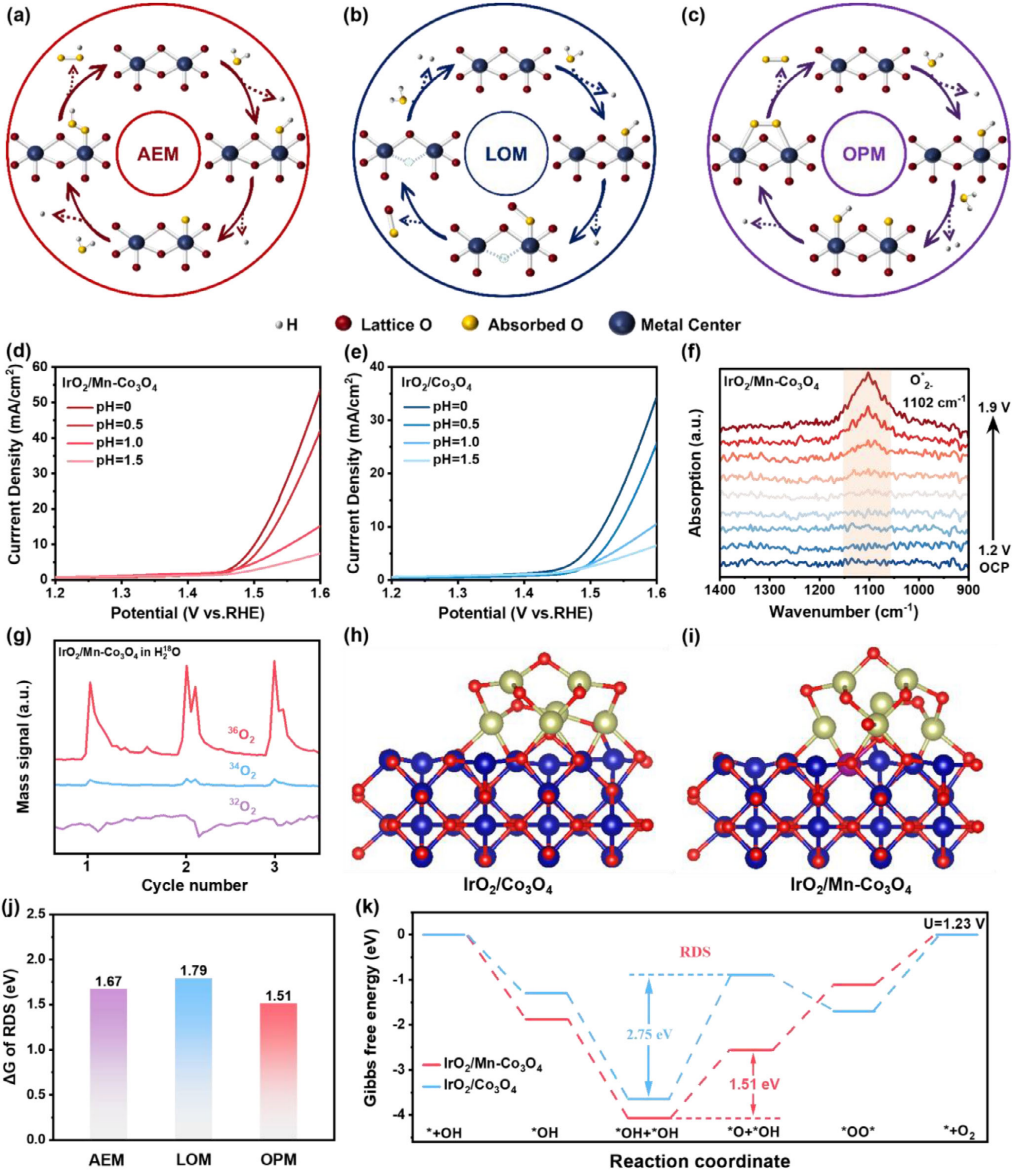
(a–c) Schematic illustration of simplified OER mechanisms: AEM, LOM, and OPM. (d‐e) LSV curves for IrO_2_/Mn‐Co_3_O_4_ and IrO_2_/Co_3_O_4_ in H_2_SO_4_ at different pH values. (f) In situ ATR‐FTIR spectra results of IrO_2_/Mn‐Co_3_O_4_ and IrO_2_/Co_3_O_4_. (g) DEMS signals of O_2_ products for IrO_2_/Mn‐Co_3_O_4_ in the electrolyte using H_2_^18^O as the solvent during three times of LSV. (h, i). Calculated models of IrO_2_/Mn‐Co_3_O_4_ catalyst and Mn‐Co_3_O_4_ structure. (j) The determined Δ*G* of RDS via the AEM, LOM, and OPM pathway in different models. (k) Gibbs free energy illustration by IrO_2_/Mn‐Co_3_O_4_ and IrO_2_/Co_3_O_4_ catalysts during the OER process by OPM pathways. The experimental data in (d–g) are representative of three independent experiments (n = 3).

The incorporation of Mn in Co_3_O_4_ not only drastically enhances the catalyst's long‐term stability, but also reduces the required overpotential. Electrocatalytic OER on metal oxides proceeds via three main pathways: the conventional adsorbate evolution mechanism (AEM) involving *OH, *O, and *OOH intermediates; the lattice‐oxygen mediated mechanism (LOM), which bypasses OOH by engaging lattice oxygen but risks structural collapse in acid; and the oxide‐path mechanism (OPM), where adjacent M─O species directly couple to O─O without extracting lattice oxygen, thus preserving integrity (Figure 5a–c). Unlike LOM, which accelerates M─O bond cleavage and catalyst degradation in acidic media, OPM offers fast kinetics without compromising structure. To unambiguously assign the active pathway in IrO_2_/Mn‐Co_3_O_4_, we performed pH‐dependent kinetics, in situ ATR‐FTIR, and operando DEMS with isotope labeling [27, 52, 53, 54, 55, 56].

First, pH‐dependent OER kinetics (Figure 5d,e and Figure S21) were systematically evaluated across a pH range of 0 to 1.5. The IrO_2_/Mn‐Co_3_O_4_ catalyst exhibited a pronounced pH‐dependent characteristic, reflected by a larger ρ_RHE_ value (16.45) compared to IrO_2_/Co_3_O_4_ (9.11), excluding the AEM mechanism for both catalysts [57]. To further validate the formation of oxygen‐related intermediates, operando synchrotron Fourier‐transform infrared (FTIR) spectroscopy was conducted under acidic OER conditions. As depicted in Figure 5f, an absorption peak in IrO_2_/Mn‐Co_3_O_4_ at 1102 cm^−1^ emerged distinctly at potentials above 1.6 V (Figure 5f), attributed to the superoxo‐like species (*O_2_^2−^) [32, 58, 59]. Similarly, the undoped IrO_2_/Co_3_O_4_ also exhibited a characteristic peak at the same wavenumber (Figure S22). The gradual increase in peak intensity with increasing potential indicates the progressive accumulation of reactive intermediates, which also support the LOM or OPM mechanism.

Operando differential electrochemical mass spectrometry (DEMS) with in situ isotope labeling was employed to elucidate the oxygen evolution mechanism on IrO_2_/Mn‐Co_3_O_4_, using 0.5 m H_2_SO_4_ in H_2_^18^O as the electrolyte. For IrO_2_/Mn‐Co_3_O_4_, the detection of ^32^O_2_, ^34^O_2_, and ^36^O_2_ signatures is characteristic of the OPM (Figure 5g) [27]. Notably, the ^36^O_2_ signal intensity exceeds that of ^32^O_2_ and ^34^O_2_ by two and three orders of magnitude, respectively. This overwhelming dominance of ^36^O_2_ unequivocally indicates that the oxygen product primarily originates from two ^18^O atoms in the isotope‐labeled electrolyte, thereby categorically excluding the LOM.

To further validate the advantages of OPM and probe the origin of enhanced activity via Mn doping in support, we calculated the Gibbs free energies for IrO_2_/Co_3_O_4_ (Figure 5h) and IrO_2_/Mn‐Co_3_O_4_ (Figure 5i) under OPM, AEM, and LOM pathways. Figure 5j presents the Gibbs free energy changes for the rate‐determining steps of IrO_2_/Mn‐Co_3_O_4_ across the three mechanisms. For OPM, the rate‐determining step is *OH+*OH → *O+*OH with an energy barrier of 1.51 eV, lower than those of AEM (1.65 eV, [*O → *OOH]) and LOM (1.79 eV, [*O+H → V_o_+O_2_]), confirming the superiority of OPM over LOM and AEM (Figures S23 and S24). Figure 5k further compares the Gibbs free energy changes of IrO_2_/Co_3_O_4_ and IrO_2_/Mn‐Co_3_O_4_. The Mn‐modified Ir sites exhibit stronger binding of oxygenated intermediates, ensuring that after OH deprotonation, the adsorbate remains coordinated as Ir─O. At optimal Ir─Ir separations, these adjacent Ir‐O species directly couple to form O‐O bonds via the oxide‐path mechanism. As a result, the energy barrier of RDS reduces from 2.75 to 1.51 eV, leading to the enhanced OER activity of the Mn‐doped sample. The doping effect of Mn on OER activity can be further explained by DOS. Compared with the d‐band center of Ir supported on pure Co_3_O_4_, the d‐band center of Ir on the Mn‐modified surface is significantly higher (Table S6). To further identify the primary active sites, the adsorption energies of *OH (ΔG_*OH_) on Ir and Co sites were calculated (Figure S25). The Ir sites exhibit a significantly stronger adsorption affinity (ΔG_*OH_ = ‐0.989 eV) compared to the Co sites (ΔG_*OH_ = −0.112 eV). This significant energy difference confirms that the OER process preferentially initiates at the Ir centers, while the Mn‐modified support primarily serves to tune the electronic structure of the active site.

**FIGURE 6:**
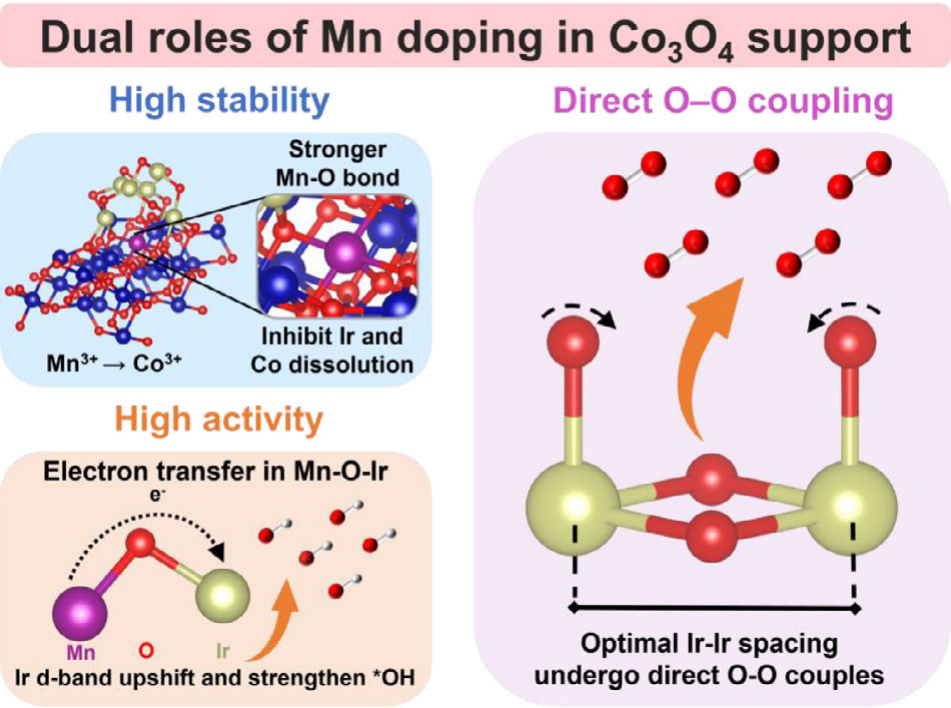
The summarized dual roles of Mn dopants in Co_3_O_4_ for enhancing the activity and stability of low‐loading IrO_2_/Co_3_O_4_ catalysts.

The effect of Mn doping on the OER activity can be summarized as below: Mn doping primarily enhances the OER activity of the catalyst through electronic modulation. The electron transfer effect within the Mn‐O‐Ir structure enriches the electron density of IrO_2_, shifting the Ir d‐band center upward. This stronger *OH binding ensures that, upon deprotonation, the adsorbate remains as Ir─O, and at optimal Ir─Ir distances these adjacent Ir─O species directly couple (oxide‐path mechanism), thereby boosting overall OER activity (Figure 6). It should be emphasized that this exceptional durability is not dictated by a single isolated factor. Rather, the suppression of Co dissolution provides a robust macroscopic structural foundation, while the strengthened Mn‐O‐Ir interfacial interaction affords direct atomic‐level electronic protection against Ir over‐oxidation. These two factors act synergistically to deliver the 6‐fold improvement in the operational lifespan.

## 3 Summary

In summary, this work demonstrates that Mn doping provides an effective dual‐regulation strategy for tuning the structural and electronic chemistry of Co_3_O_4_ support, thereby overcoming the intertwined challenges of activity and stability in low‐Ir acidic OER catalysts. Compared to the undoped counterpart, IrO_2_/Mn‐Co_3_O_4_ exhibits 6 times higher stability and a significantly reduced overpotential of 51 mV. Structural stabilization arises from the substitution of Mn^3+^ into octahedral Co sites, forming highly covalent Mn─O bonds that anchor the spinel lattice and suppress both Co/Ir dissolution and lattice collapse, as evidenced by in situ Raman, XAFS, XPS, and corroborated by DOS, CBM, and ELF analyses. Simultaneous electronic activation occurs via Mn─O─Ir electron donation, which shifts the Ir d‐band center upward, optimizing *OH adsorption so that, after deprotonation, Ir─O species remain intact and, at ideal Ir─Ir separations, undergo direct O─O coupling through the oxide‐path mechanism. These atomic‐scale insights reveal that Mn‐induced support stabilization and Ir electronic activation act synergistically to drive efficient and durable acidic OER, providing a clear design blueprint for the development of high‐performance, acid‐stable, and low‐iridium catalysts for sustainable hydrogen production. Beyond improving the long‐term electrocatalytic durability, the significantly suppressed Ir and Co dissolution via Mn doping also substantially lowers the potential ecotoxicological burden of the catalyst on aquatic environments [60].

Crucially, the Mn‐doped Co_3_O_4_ support functions as a universal and robust platform capable of stabilizing various active phases—from Ir/Ru clusters to dual‐atom pairs—by synergistically modulating interfacial electronic states and reinforcing the spinel lattice against acidic corrosion [15, 20, 33, 61, 62]. This strategy provides a generalizable blueprint for the rational design of high‐performance, acid‐stable electrocatalysts for industrial‐scale PEM water electrolysis.

## Conflicts of Interest

The authors declare no conflicts of interest.

## Supporting information




**Supporting File**: advs75667‐sup‐0001‐SuppMat.docx.

## Data Availability

The data that support the findings of this study are available from the corresponding author upon reasonable request.

## References

[1] Q. Wang , Y. Cheng , H. B. Tao , et al., “Long‐Term Stability Challenges and Opportunities in Acidic Oxygen Evolution Electrocatalysis,” Angewandte Chemie International Edition 62 (2023): 202216645, 10.1002/anie.202216645. 36546885

[2] Z. Liu , G. Ma , X. Cao , et al., “Dual‐Ion Engineering of Fe‐Doped Ni_3_(OH)_4_(NO_3_)_2_ for Cl^−^‐Resistant Oxygen Evolution Reaction in Alkaline Medium,” AIChE Journal 72 (2026): 70137, 10.1002/aic.70137.

[3] R. Wan , T. Yuan , L. Wang , B. Li , M. Liu , and B. Zhao , “Earth‐Abundant Electrocatalysts for Acidic Oxygen Evolution,” Nature Catalysis 7 (2024): 1288–1304, 10.1038/s41929-024-01266-6.

[4] R.‐T. Liu , Z.‐L. Xu , F.‐M. Li , et al., “Recent Advances in Proton Exchange Membrane Water Electrolysis,” Chemical Society Reviews 52 (2023): 5652–5683, 10.1039/D2CS00681B. 37492961

[5] X. Cao , Y. Kang , T. Jiang , et al., “Facet‐Selective Etching by Pyridazine Toward Robust Ruthenium‐based Oxygen Evolution Catalysts,” Nano Research 18 (2025): 94907315, 10.26599/NR.2025.94907315.

[6] L. Kong , D. Zhou , K. Tian , et al., “Dual‐Site Engineering Promotes Oxygen Evolution Reaction of Acidic Water Electrolysis over RuO_2_ ,” Small 21 (2025): 2505346, 10.1002/smll.202505346. 40719499

[7] C. Spöri , J. T. H. Kwan , and A. Bonakdarpour , “Towards a Common Fundamental Understanding and Mitigation of Catalyst Degradation,” Angewandte Chemie International Edition 56 (2017): 5994–6021. 27805788 10.1002/anie.201608601

[8] Z.‐Y. Wu , F.‐Y. Chen , B. Li , et al., “Non‐iridium‐based Electrocatalyst for Durable Acidic Oxygen Evolution Reaction in Proton Exchange Membrane Water Electrolysis,” Nature Materials 22 (2023): 100–108, 10.1038/s41563-022-01380-5. 36266572

[9] F. Xie , Y. Du , M. Lu , S. Yan , and Z. Zou , “Thermal‐stimulated Spin Disordering Accelerates Water Electrolysis,” Energy & Environmental Science 18 (2025): 1972–1983, 10.1039/D4EE04597A.

[10] Z. Liu , G. Ma , X. Zhu , et al., “Anion Effect in Electrocatalysts for Oxygen Evolution Reaction and Small Organic Molecule Oxidation,” ACS Energy Letters 11 (2026): 1514–1537, 10.1021/acsenergylett.5c04078.

[11] L. Jiang , B. Duan , S. Lin , et al., “High‐entropy Chalcogenides via Ambient and Scalable Synthesis for Efficient OER Catalysis,” Nano Energy 148 (2026): 111634, 10.1016/j.nanoen.2025.111634.

[12] L. Wu , B. Zhao , W. Huang , et al., “Dynamic Chromium Dopant Promotes Interfacial Water Activation on Cobalt Spinel Oxide for Efficient Oxygen Evolution in Acid,” Nature Communications 17 (2026): 2598, 10.1038/s41467-026-69124-6. PMC13002916 41667460

[13] Y. Wen , P. Chen , L. Wang , et al., “Stabilizing Highly Active Ru Sites by Suppressing Lattice Oxygen Participation in Acidic Water Oxidation,” Journal of the American Chemical Society 143 (2021): 6482–6490, 10.1021/jacs.1c00384. 33891414

[14] L. Huang , X. Zhao , J. Kang , et al., “Molybdenum‐doping Triggers Lattice Oxygen Oxidation for Enhanced Acidic Oxygen Evolution Reaction,” Journal of Alloys and Compounds 1056 (2026): 186596, 10.1016/j.jallcom.2026.186596.

[15] L. Jiang , C. Wang , Z. Shao , and G. Jia , “Synthesis and Catalytic Behaviour of IrO_2_ Nanostructures in Acidic Water Oxidation,” Advanced Nanocomposites 3 (2026): 149–179, 10.1016/j.adna.2026.02.001.

[16] X. Han , T. Mou , S. Kang , et al., “Enhancing Acidic Oxygen Evolution Activity by Controlling Oxidation State of Iridium,” Angewandte Chemie International Edition 64 (2025): 202507468, 10.1002/anie.202507468. 40495316

[17] Y. Zhu , F. Guo , Q. Wei , et al., “Engineering the Metal/Oxide Interfacial O‐Filling Effect to Tailor Oxygen Spillover for Efficient Acidic Water Oxidation,” Advanced Functional Materials 35 (2025): 2421354, 10.1002/adfm.202421354.

[18] J. Kang , X. Wang , S. Möhle , et al., “Synthesis, Molecular Structure, and Water Electrolysis Performance of TiO_2_‐Supported Raney‐IrOx Nanoparticles for the Acidic Oxygen Evolution Reaction,” ACS Catalysis 15 (2025): 5435–5446, 10.1021/acscatal.4c06385.

[19] X. Han , T. Mou , A. Islam , et al., “Theoretical Prediction and Experimental Verification of IrO_x_ Supported on Titanium Nitride for Acidic Oxygen Evolution Reaction,” Journal of the American Chemical Society 146 (2024): 16499–16510, 10.1021/jacs.4c02936. 38859684

[20] J. Yuan , G. Li , Y. Tan , et al., “Coupling Electron‐buffered Dual Single‐atom Pairs to Unlock Exceptional Industrial‐grade PEM Water Electrolysis,” Joule (2026): 102425, 10.1016/j.joule.2026.102425.

[21] Y. Tang , N.‐N. Liang , Y. Li , et al., “Confined Kirkendall Strain Engineering Promotes Oxygen Evolution on Co_3_O_4_ for Proton Exchange Membrane Water Electrolyzer,” Advanced Energy Materials 16 (2026): 06692, 10.1002/aenm.202506692.

[22] Z. Lin , T. Wang , and Q. Li , “Designing Active and Stable Ir‐based Catalysts for the Acidic Oxygen Evolution Reaction,” Industrial Chemistry & Materials 1 (2023): 299–311, 10.1039/D3IM00070B.

[23] H. Wang , X. Li , G. Zhang , et al., “Recent Progress in Balancing the Activity, Durability, and Low Ir Content for Ir‐Based Oxygen Evolution Reaction Electrocatalysts in Acidic Media,” Small 21 (2025): 2410407, 10.1002/smll.202410407. 39711255

[24] S. Niu , X.‐P. Kong , S. Li , et al., “Low Ru Loading RuO_2_/(Co, Mn)_3_O_4_ Nanocomposite with Modulated Electronic Structure for Efficient Oxygen Evolution Reaction in Acid,” Applied Catalysis B: Environmental 297 (2021): 120442, 10.1016/j.apcatb.2021.120442.

[25] A. Li , S. Kong , K. Adachi , et al., “Atomically Dispersed Hexavalent Iridium Oxide from MnO_2_ Reduction for Oxygen Evolution Catalysis,” Science 384 (2024): 666–670, 10.1126/science.adg5193. 38723092

[26] S. She , C. Chen , K. Fan , et al., “Optimizing the Ru Catalyst–Support Interaction via Tunnel Size of MnO_2_ Support for Enhanced Acidic Water Oxidation,” Journal of the American Chemical Society 147 (2025): 24392–24402, 10.1021/jacs.5c02857. 40609049

[27] C. Lin , J.‐L. Li , X. Li , et al., “In‐situ Reconstructed Ru Atom Array on α‐MnO_2_ with Enhanced Performance for Acidic Water Oxidation,” Nature Catalysis 4 (2021): 1012–1023, 10.1038/s41929-021-00703-0.

[28] D. Liu , Y. Tang , K. Liu , et al., “Mn Doping Induced Electronic Modulation of Self‐supported NiFe Layered Double Hydroxides for Oxygen Evolution Reaction,” New Journal of Chemistry 49 (2025): 6723–6730, 10.1039/D4NJ05317F.

[29] R. Wei , D. Li , P. Zhou , et al., “Unraveling the Formation Kinetics of the First Intermediate in the Oxygen Evolution Reaction on MnOx with Different Electron Configurations,” Journal of the American Chemical Society 147 (2025): 23473–23481, 10.1021/jacs.4c18273. 40583328

[30] A. Li , S. Kong , C. Guo , et al., “Enhancing the Stability of Cobalt Spinel Oxide towards Sustainable Oxygen Evolution in Acid,” Nature Catalysis 5 (2022): 109–118, 10.1038/s41929-021-00732-9.

[31] L. Chong , G. Gao , J. Wen , et al., “La‐ and Mn‐doped Cobalt Spinel Oxygen Evolution Catalyst for Proton Exchange Membrane Electrolysis,” Science 380 (2023): 609–616, 10.1126/science.ade1499. 37167381

[32] H. Zhao , L. Zhu , J. Yin , et al., “Stabilizing Lattice Oxygen through Mn Doping in NiCo_2_O_4‐δ_ Spinel Electrocatalysts for Efficient and Durable Acid Oxygen Evolution,” Angewandte Chemie International Edition 63 (2024): 202402171, 10.1002/anie.202402171. 38494450

[33] S. Zhu , R. Yang , H. J. W. Li , et al., “Reconstructing Hydrogen‐Bond Network for Efficient Acidic Oxygen Evolution,” Angewandte Chemie International Edition 63 (2024): 202319462, 10.1002/anie.202319462. 38286750

[34] A. Kumar , M. Gil‐Sepulcre , J. Lee , et al., “Iridium Single‐Atom‐Ensembles Stabilized on Mn‐Substituted Spinel Oxide for Durable Acidic Water Electrolysis,” Advanced Materials 36 (2024): 2401648, 10.1002/adma.202401648. 39318088

[35] J. Shan , C. Ye , S. Chen , et al., “Short‐Range Ordered Iridium Single Atoms Integrated into Cobalt Oxide Spinel Structure for Highly Efficient Electrocatalytic Water Oxidation,” Journal of the American Chemical Society 143 (2021): 5201–5211, 10.1021/jacs.1c01525. 33764061

[36] W. Xie , S. J. H. Ong , Z. Shen , et al., “Critical Role of Tetrahedral Coordination in Determining the Polysulfide Conversion Efficiency on Spinel Oxides,” Journal of the American Chemical Society 147 (2025): 988–997, 10.1021/jacs.4c14263. 39780387

[37] D. Li , D. Xu , Y. Pei , Q. Zhang , Y. Lu , and B. Zhang , “Isolated Octahedral Pt‐Induced Electron Transfer to Ultralow‐Content Ruthenium‐Doped Spinel Co_3_O_4_ for Enhanced Acidic Overall Water Splitting,” Journal of the American Chemical Society 146 (2024): 28728–28738, 10.1021/jacs.4c07089. 39268752

[38] X. Li , X. Ma , D. Su , et al., “Direct Visualization of the Jahn–Teller Effect Coupled to Na Ordering in Na_5/8_MnO_2_ ,” Nature Materials 13 (2014): 586–592, 10.1038/nmat3964. 24836735

[39] J. Han , H. Wang , Y. Wang , et al., “Lattice Oxygen Activation through Deep Oxidation of Co_4_N by Jahn–Teller–Active Dopants for Improved Electrocatalytic Oxygen Evolution,” Angewandte Chemie International Edition 63 (2024): 202405839, 10.1002/anie.202405839. 38801294

[40] S. Czioska , A. Boubnov , D. Escalera‐López , et al., “Increased Ir–Ir Interaction in Iridium Oxide during the Oxygen Evolution Reaction at High Potentials Probed by Operando Spectroscopy,” ACS Catalysis 11 (2021): 10043–10057, 10.1021/acscatal.1c02074.

[41] S. Ma , K. Wang , M. Rafique , et al., “Reconstruction of Ferromagnetic/Paramagnetic Cobalt‐Based Electrocatalysts under Gradient Magnetic Fields for Enhanced Oxygen Evolution,” Angewandte Chemie International Edition 63 (2024): 202412821, 10.1002/anie.202412821. 39105426

[42] Y. Dai , Q. Tang , Y. Hu , et al., “Structurally Disordered CoSx–Co(OH)_2_ Heterointerface for Boosting Alkaline Hydrogen Evolution Reaction,” Journal of Colloid and Interface Science 713 (2026): 140148, 10.1016/j.jcis.2026.140148. 41747581

[43] Y. Zhu , J. Wang , T. Koketsu , et al., “Iridium Single Atoms Incorporated in Co_3_O_4_ Efficiently Catalyze the Oxygen Evolution in Acidic Conditions,” Nature Communications 13 (2022): 7754, 10.1038/s41467-022-35426-8. PMC9751110 36517475

[44] C. Wei , R. R. Rao , J. Peng , et al., “Recommended Practices and Benchmark Activity for Hydrogen and Oxygen Electrocatalysis in Water Splitting and Fuel Cells,” Advanced Materials 31 (2019): 1806296, 10.1002/adma.201806296. 30656754

[45] B. Guo , H. Huo , Q. Zhuang , et al., “Iron Oxyhydroxide: Structure and Applications in Electrocatalytic Oxygen Evolution Reaction,” Advanced Functional Materials 33 (2023): 2300557, 10.1002/adfm.202300557.

[46] L. Xu , Y. Xu , B. Xia , et al., “Surface Single Atom Alloys for Alkaline Hydrogen Evolution Reaction,” Advanced Materials 37 (2025): 2502989, 10.1002/adma.202502989. 40259633

[47] A. Moysiadou , S. Lee , C.‐S. Hsu , H. M. Chen , and X. Hu , “Mechanism of Oxygen Evolution Catalyzed by Cobalt Oxyhydroxide: Cobalt Superoxide Species as a Key Intermediate and Dioxygen Release as a Rate‐Determining Step,” Journal of the American Chemical Society 142 (2020): 11901–11914, 10.1021/jacs.0c04867. 32539368

[48] N. Yao , G. Wang , H. Jia , et al., “Intermolecular Energy Gap‐Induced Formation of High‐Valent Cobalt Species in CoOOH Surface Layer on Cobalt Sulfides for Efficient Water Oxidation,” Angewandte Chemie International Edition 61 (2022): 202117178, 10.1002/anie.202117178. 35037704

[49] Z. Chen , L. Cai , X. Yang , et al., “Reversible Structural Evolution of NiCoOxHy during the Oxygen Evolution Reaction and Identification of the Catalytically Active Phase,” ACS Catalysis 8 (2018): 1238–1247, 10.1021/acscatal.7b03191.

[50] W. Zhu , F. Yao , K. Cheng , et al., “Direct Dioxygen Radical Coupling Driven by Octahedral Ruthenium–Oxygen–Cobalt Collaborative Coordination for Acidic Oxygen Evolution Reaction,” Journal of the American Chemical Society 145 (2023): 17995–18006, 10.1021/jacs.3c05556. 37550082

[51] J. Zhou , Y. Wang , X. Su , et al., “Electrochemically Accessing Ultrathin Co (oxy)‐hydroxide Nanosheets and Operando Identifying Their Active Phase for the Oxygen Evolution Reaction,” Energy & Environmental Science 12 (2019): 739–746, 10.1039/C8EE03208D.

[52] M. Wohlfahrt‐Mehrens and J. Heitbaum , “Oxygen Evolution on Ru and RuO_2_ Electrodes Studied Using Isotope Labelling and on‐line Mass Spectrometry,” Journal of Electroanalytical Chemistry and Interfacial Electrochemistry 237 (1987): 251–260, 10.1016/0022-0728(87)85237-3.

[53] L. Li , P. Wang , Q. Shao , and X. Huang , “Recent Progress in Advanced Electrocatalyst Design for Acidic Oxygen Evolution Reaction,” Advanced Materials 33 (2021): 2004243, 10.1002/adma.202004243. 33749035

[54] G. Ma , H. Huo , Y. Hu , et al., “Engineering Acid‐Stable OER Electrocatalysts: Recent Advances in Main‐Group Element Doping of Ir‐ and Ru‐Based Oxides,” ACS Catalysis 16 (2026): 1871–1893, 10.1021/acscatal.5c08506.

[55] Q. Ji , B. Tang , X. Zhang , et al., “Operando Identification of the Oxide Path Mechanism with Different Dual‐active Sites for Acidic Water Oxidation,” Nature Communications 15 (2024): 8089, 10.1038/s41467-024-52471-7. PMC11405856 39284800

[56] C.‐T. He , L.‐H. Yu , H. Liu , et al., “Post‐Oxidation of All‐organic Electrocatalysts to Promote O−O Coupling in Water Oxidation,” Nature Communications 16 (2025): 4389, 10.1038/s41467-025-59771-6. PMC12069523 40355445

[57] A. K. Tomar , U. N. Pan , N. H. Kim , and J. H. Lee , “Enabling Lattice Oxygen Participation in a Triple Perovskite Oxide Electrocatalyst for the Oxygen Evolution Reaction,” ACS Energy Letters 8 (2023): 565–573, 10.1021/acsenergylett.2c02617.

[58] Y. Hao , S.‐F. Hung , C. Tian , et al., “Polarized Ultrathin BN Induced Dynamic Electron Interactions for Enhancing Acidic Oxygen Evolution,” Angewandte Chemie International Edition 63 (2024): 202402018, 10.1002/anie.202402018. 38390636

[59] R. R. Rao , M. J. Kolb , and L. Giordano , “Operando Identification of Site‐dependent Water Oxidation Activity on Ruthenium Dioxide Single‐crystal Surfaces,” Nature Catalysis 3 (2020): 516–525.

[60] Z. Liu , Y. Gao , X. Guo , and S. Li , “Assessing the Neuro‐ and Immunotoxicity of Dissolved Ru^3+^ from Proton Exchange Membrane Electrolyzers by Zebrafish Models,” ACS Applied Materials & Interfaces 18 (2026): 5229–5242, 10.1021/acsami.5c20368. 41537674

[61] Z. Li , Y. Liu , J. Hu , et al., “RuO _2_ Sub‐Nanocluster Decorated Co _3_ O _4_ as Efficient and pH ‐Universal Oxygen Evolution Electrocatalyst,” InfoMat 7 (2025): 70003, 10.1002/inf2.70003.

[62] R.‐Y. Fan , H. Liu , J.‐K. Ren , et al., “Ligand‐Confinement‐Induced Catalyst–Support Interface Interactions in Co_3_O_4_‐Supported RuO_2_ for Long‐Term Stable Acidic Oxygen Evolution Reaction,” ACS Sustainable Chemistry & Engineering 12 (2024): 2313–2323, 10.1021/acssuschemeng.3c06895.

