# Supplementary material for: Mn‐Induced Support Stabilization and Ir Electronic Activation Enable Acid‐Stable, Low‐Loading IrO2 Water Oxidation
Source: Adv Sci (Weinh). 2026 May 11:e75667. Online ahead of print. doi: 10.1002/advs.75667 (PMC13336039; doi:10.1002/advs.75667)
Supplement: Supplementary file 1 — Supporting File: advs75667‐sup‐0001‐SuppMat.docx. [file ADVS-9999-e75667-s001.docx]

Supporting Information

Mn-Induced Support Stabilization and Ir Electronic Activation Enable Acid-Stable, Low-Loading IrO_2_ Water Oxidation

*Zhe Liu ^a, d, g+^, Guoxin Ma ^a+^, Shixiang Yu ^b+^, Rui Jin ^a^, Fei Wang ^a^,* *Xinxin Wen ^a*^, Mengxin Chen ^c^*, Yani Ding ^e^, Jia Liu ^f^, Xinkai Guo ^g^, Diab Khalafallah ^h^, Hassan Fouad ^i^, Xiao Ren ^b^*, Siwei Li ^a, g^**

*^a^* State Key Laboratory of Fluorine & Nitrogen Chemicals, School of Chemical Engineering and Technology, Xi’an Jiaotong University, Xi’an 710049, China

*^b^* Beijing National Laboratory for Molecular Engineering, College of Chemistry and Molecular Engineering, Peking University, 100871, China

*^c^* Institute of Sustainability for Chemicals, Energy and Environment (ISCE2), Agency for Science, Technology and Research (A*STAR), 1 Pesek Road, Jurong Island, Singapore 627833.

*^d^* Department of Physiology and Pathophysiology, School of Basic Medical Sciences, Health Science Center, Xi’an Jiaotong University, Xi’an 710061, China

*^e^* International Research Center for Renewable Energy, State Key Laboratory of Multiphase Flow in Power Engineering, School of Energy and Power Engineering, Xi’an Jiaotong University, Xi’an, Shaanxi, 710049 P.R. China

^f^ Instrumental Analysis Center, Xi’an Jiaotong University, Xi’an 710049, China

^g^ Institute of Electronic and Information Engineering of UESTC in Guangdong, Dongguan 523808, PR China

^h^ Mechanical Design and Materials Department, Faculty of Energy Engineering, Aswan University, Aswan 81521, Egypt

^i^ Health Sciences Department, College of Applied Studies, King Saud University, Riyadh, 12642, Saudi Arabia

*^+^* These authors contribute equally to this work

Corresponding author. E-mail: [xinxinxin@stu.xjtu.edu.cn](mailto:xinxinxin@stu.xjtu.edu.cn) , [chenmengxin_hit@163.com](mailto:chenmengxin_hit@163.com) , [renxiao_@pku.edu.cn](mailto:renxiao_@pku.edu.cn) , [lisiwei@xjtu.edu.cn](mailto:lisiwei@xjtu.edu.cn)

**Experimental Section**

***Chemicals and Materials*:** Cobalt (II) nitrate hexahydrate (Co(NO_3_)_2_·6H_2_O, ≥98.0%, Sinopharm Chemical Reagent Co., Ltd.), 2-methylimidazole (C_4_H_6_N_2_, ≥98.0%, Aladdin), Iridium (III) chloride trihydrate (IrCl_3_·xH_2_O, ≥99%, Aladdin), potassium permanganate (KMnO_4_, ≥99.5%, Sinopharm), iridium dioxide (IrO_2_, ≥99.9%, Aladdin). All reagents were purchased and used directly without any purification. Carbon cloth (CC) was purchased from CeTech Co., Ltd. and cut into pieces of 2 × 2 cm^2^ for use. The CC was cleaned with acetone, ethanol, and deionized water with the assistance of ultrasonication for 30 min, respectively. Then, the CC pieces were further ultrasound-treated for 4 h in concentrated nitric acid and finally washed with deionized water in neutral environment for later use.

**Synthesis of IrO_2_/Mn-Co_3_O_4_, IrO_2_/Co_3_O_4_, Mn-Co_3_O_4_ and Co_3_O_4_**

***Synthesis of ZIF-67 precursor:*** ZIF-67 precursor was prepared as the method reported by our early work.

***Synthesis of Mn-ZIF-67*:** A solution of 0.002 g KMnO_4_ in 8 mL deionized water was prepared under stirring. The ZIF-67 was immersed in this solution and reacted in an ice bath for 30 min. After the reaction, the sample was washed repeatedly with deionized water and ethanol, followed by drying to obtain Mn-ZIF-67.

***Synthesis of Ir/Mn-ZIF-67:*** 2 mmol/L IrCl_3_ aqueous solution of was prepared and immersed Mn-ZIF-67 in 5 mL IrCl_3_ solution. Let it stand at room temperature for 24 hours. The sample was then rinsed with deionized water and dried to yield Ir/Mn-ZIF-67.

***Synthesis of*** ***IrO_2_/Mn-Co_3_O_4_:*** Ir/Mn-ZIF-67 was placed in a muffle furnace and annealed in air at a heating rate of 2 °C/min to 300 °C, followed by holding at this temperature for 4 h. After cooling to room temperature naturally, the final product, denoted as IrO_2_/Mn-Co_3_O_4_, was obtained.

***Synthesis of*** ***IrO_2_/Co_3_O_4_:*** For comparison, ZIF-67 (without Mn doping) was subjected to the same ion exchange and annealing procedures (steps 4-5) to prepare IrO_2_/Co_3_O_4_.

***Synthesis of Mn-Co_3_O_4_:*** For comparison, Mn-ZIF-67 (without ion exchange) was subjected to same annealing procedures.

***Synthesis of Co_3_O_4_:*** ZIF-67 annealed in air at a heating rate of 2 °C/min to 300 °C, followed by holding at this temperature for 4 h.

**Characterizations of Catalysts**

The X-ray diffraction (XRD) patterns of the samples were recorded on a Rigaku D/MAXRC X-ray diffractometer at 45 mA and 40 kV using a Cu target as the anticathode. X-ray photoelectron spectroscopy (XPS) data of the catalysts were obtained using an ESCALAB 250Xi system with Al Kα radiation as the source (hν = 1486.6 eV). Scanning electron microscopy (SEM) images were collected on a Phenom ProX from the Netherlands. Special Aberration Corrected Transmission Electron Microscope (STEM) images of the samples for lattice structure analyses were recorded using a JEOL JEM-ARM300F2 transmission electron microscope operating at an accelerating voltage of 200 kV. Energy Dispersive X-ray spectroscopy (EDX) was used to collect the elemental mapping of all samples with an EDX detector (JEOL JEM-ARM300F2 with a 100 mm^2^ windowless silicon drift detector) attached to the TEM. Raman measurements were performed on a Laser Raman Spectrometer (Shaanxi Longrun International Trade Co., LTD) with an excitation wavelength of 532 nm. The fourier transform infrared spectroscopy (FT-IR) was obtained with Tensor-27, Germany, Bruker system. And the actual metal content of IrO_2_/Mn-Co_3_O_4_ and IrO_2_/Co_3_O_4_ were detected by inductively coupled plasma-atomic emission spectrometry (ICP-AES). The X-ray absorption near Edge structure (XANES) and Extended X-ray Absorption Fine Structure (EXAFS) of the sample were tested at the XAFCA beamline of the Singapore Synchrotron Light Source (SSLS) within transmission mode. The storage ring of SSLS operated at *E* = 700 MeV, *I*_max_ = 200 mA.

**Electrochemical Measurements**

All electrochemical measurements were carried out on the electrochemical workstation CHI660E (Chenhua, Shanghai, China) using a standard three-electrode system in 0.5 M H_2_SO_4_. Hg/Hg_2_SO_4_ was employed as the reference electrode, the catalyst as the working electrode (area= 1cm^2^), and the graphite electrode as the counter electrode. The linear sweep voltammetry curve (LSV) was recorded at a scan rate of 5 mV/s and then corrected with 85% *iR* correction. The double layer capacitance (*C*_dl_) of the catalysts was calculated by measuring the cyclic voltamperes (CV) at different scan rates (10, 20, 40, 60, 80, 100, 120, and 150 mV/s). The electrochemically active surface area (ECSA) was calculated as follows:

| *ECSA* = $\frac{\text{C}\text{dl}}{\text{40 }\text{μ}\text{F }\text{cm}^{\text{-2}}}$ |  |
| --- | --- |

Electrochemical impedance spectroscopy (EIS) was measured in the frequency range from 0.1 to 10^6^ Hz.

**Proton Exchange Membrane Water Electrolyzer (PEMWE) Measurements**

To construct the PEMWE, the prepared IrO_2_/Mn-Co_3_O_4_ catalysts was used as the anode, and commercial Pt/C was utilized as the cathode catalyst. To prepare the cathode and anode ink, catalysts were firstly dispersed to a mixture of Nafion (5%), isopropanol and distilled water. After ultrasonicated for at least 1 h in a low temperature water bath, a uniform catalyst ink can be obtained. The IrO_2_/Mn-Co_3_O_4_ catalyst and commercial Pt/C catalysts were directly air sprayed on the two sides of the as-received Nafion 212 membrane in the ultrasonic spray coating system to prepare the PEMWE with Nafion 212 membrane. The total catalyst loading for both the anode and cathode was controlled at 4 mg_cat_ cm^-2^ following loading optimization. Specifically, based on the ICP-determined iridium content (14.5 wt%), the corresponding Ir loading on the anode was approximately 0.58 mg_Ir_ cm^-2^. The PEM electrolyzers were operated at 60 °C with pure water as reactant under a flow rate of 100 mL min^-1^. All the data of PEMWE were displayed as raw data without *iR* correction.

**Theoretical Calculations**

The geometry structures and electronic structure analysis were studied by the Density Functional Theory (DFT) with the *Vienna Ab initio* Simulation Package (VASP) using the PBE exchange-correlation function. [1] A DFT-D3-BJ correction was adopted to account for *van der Waals* interactions. [2] The geometry structures were optimized with three layers of bottom metal atoms fixed in the slab model. The cutoff energy of plane-wave basis was set to 400 eV. For k-space sampling, the 4*3*1 Monkhorst-Pack grid was used in the calculations. GGA + U calculations were undertaken using the single effective parameter U_eff_ = 3.5, 3.3 and 1.0 eV which were determined for Co, Mn and Ir species in models. The convergence threshold of energy and forces were set to be 1E-5 eV and 0.02 eV Å^-1^, respectively.

In the process of data post-processing, VASPKIT and LOBSTER and VMD codes were used. ^[3,4]^

**Statistical Analysis**

All electrochemical measurements, including linear sweep voltammetry (LSV), cyclic voltammetry (CV), chronopotentiometry, and electrochemical impedance spectroscopy (EIS), were performed independently at least three times to ensure accuracy and reproducibility (n ≥ 3). The experimental data are presented as mean ± standard deviation (SD) where applicable. No data points or outliers were excluded prior to analysis. The calculations for the double-layer capacitance (*C*_dl_) and proton reaction orders were evaluated using standard linear regression, with the goodness-of-fit validated by corresponding R^2^ values. For theoretical calculations, the convergence thresholds for energy and forces were strictly set to 1 × 10^^-5^ eV and 0.02 eV Å ^-1^, respectively. Experimental data processing and plotting were conducted using Origin software, while DFT data post-processing utilized VASPKIT, LOBSTER, and VMD codes.

**
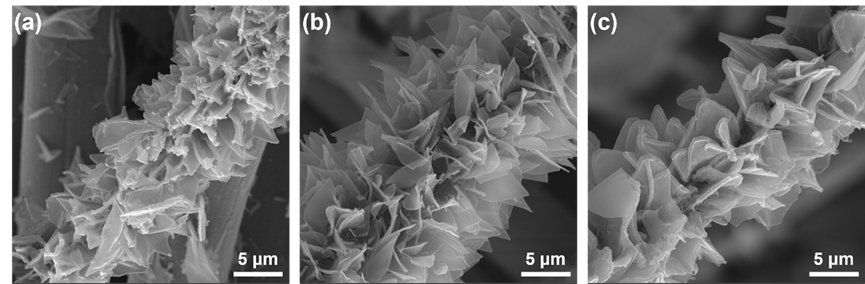
**

**Figure S1.** SEM images of (a) IrO_2_/Co_3_O_4_ (b) Mn-Co_3_O_4_ (c) Co_3_O_4_.


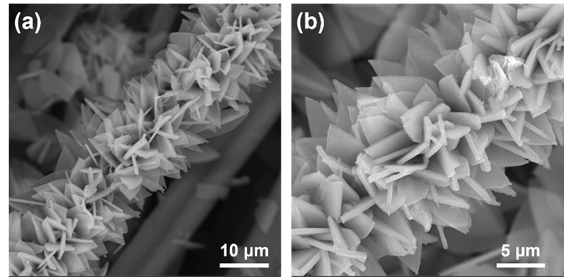


**Figure S2.** SEM images of Mn-ZIF-67.

**
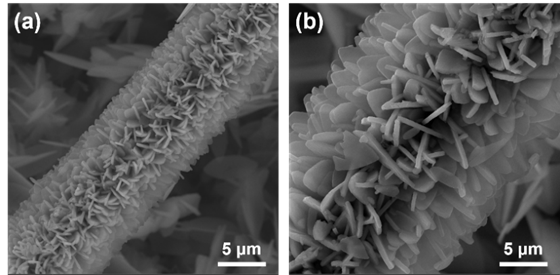
**

**Figure S3.** SEM images of ZIF-67.


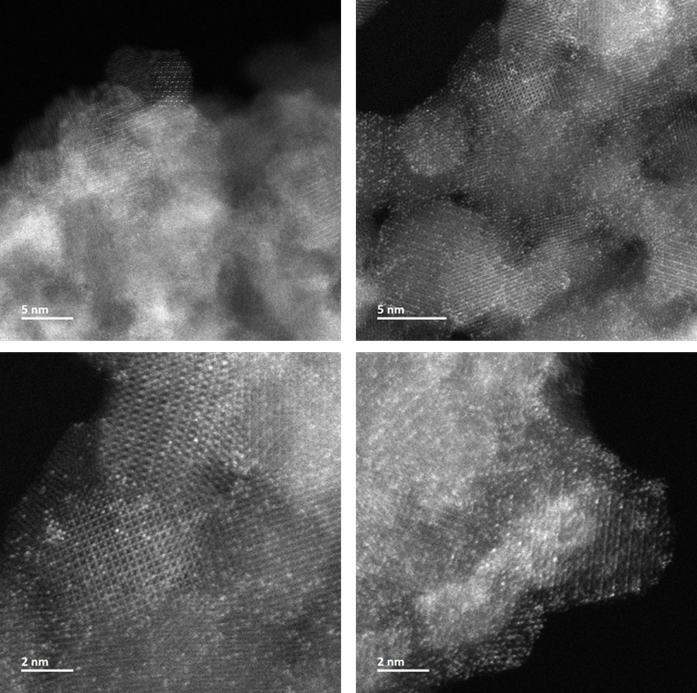


**Figure S4.** AC-HAADF-STEM images of IrO_2_/Mn-Co_3_O_4_ with different magnifications.


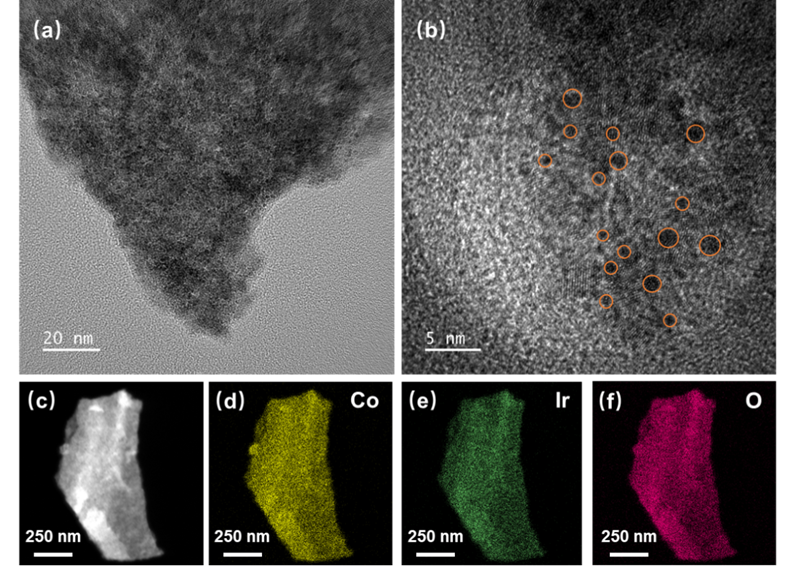


**Figure S5.** (a) TEM, (b) HRTEM, (c-f) Elements mapping images of IrO_2_/Co_3_O_4_.


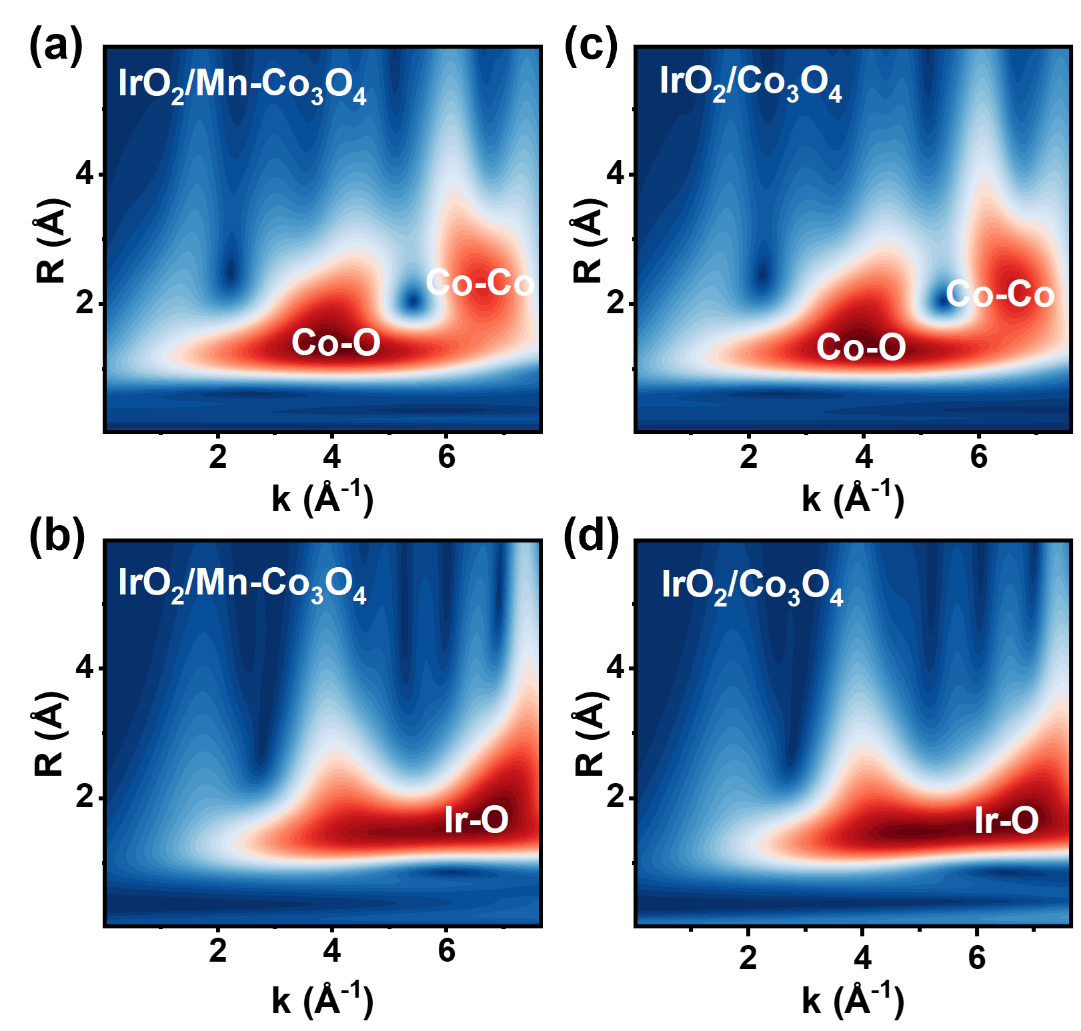


**Figure S6.** WT-EXAFS spectra of (a-b) IrO_2_/Mn-Co_3_O_4_ and (c-d) IrO_2_/Co_3_O_4_.


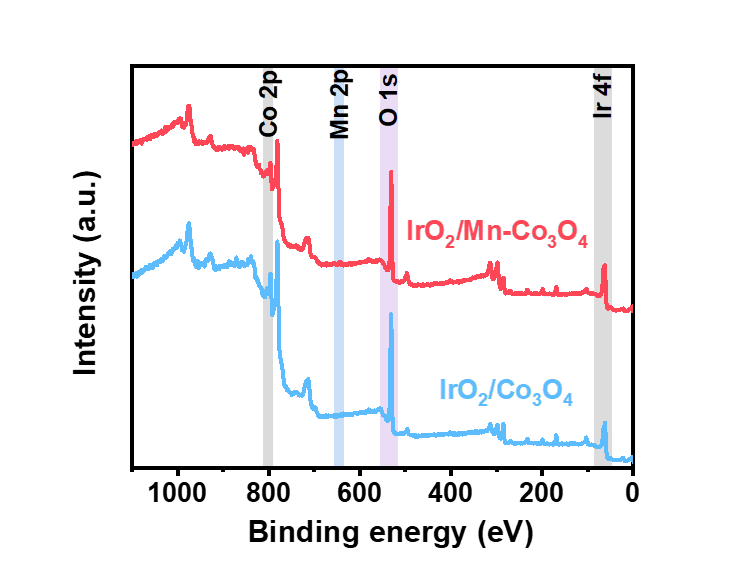


**Figure S7.** Survey XPS spectra of IrO_2_/Mn-Co_3_O_4_ and IrO_2_/Co_3_O_4_.

**
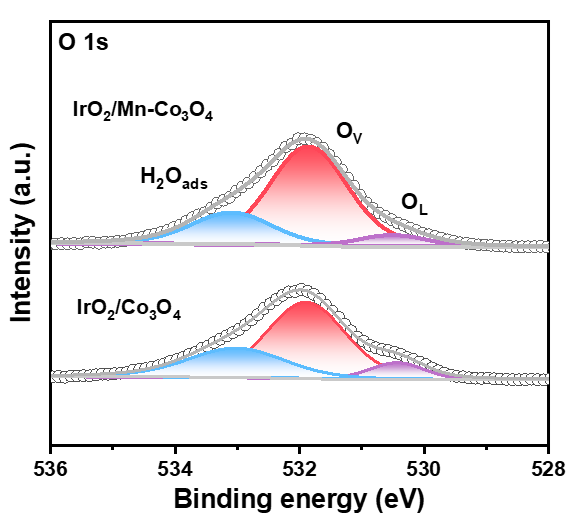
**

**Figure S8.** The XPS O 1s spectra of IrO_2_/Mn-Co_3_O_4_ and IrO_2_/Co_3_O_4_.

**
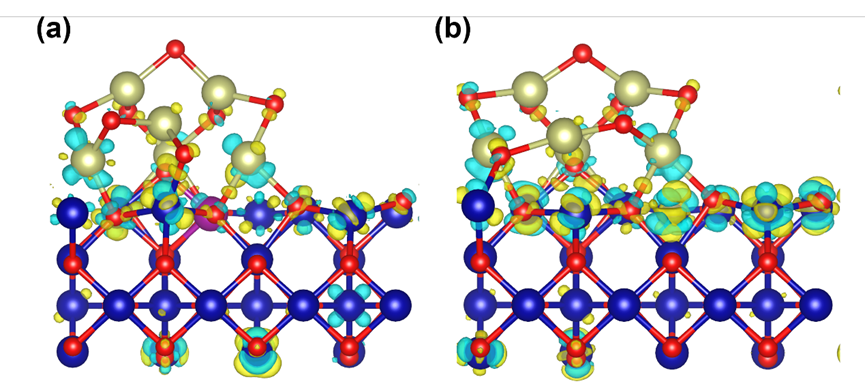
**

**Figure S9.** Differential charge density of (a) IrO_2_/Mn-Co_3_O_4_ and (b) IrO_2_/ Co_3_O_4_ (isosurface level = 0.02).


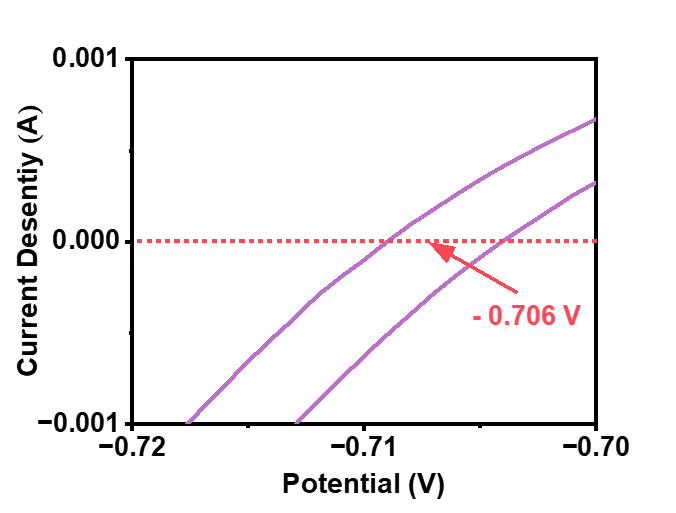


**Figure S10.** Hg/Hg_2_SO_4_ reference electrode calibration in H_2_ saturated 0.5 M H_2_SO_4_ solution.

**Figure S11.** Acidic OER LSV polarization curves of IrO_2_/Mn-Co_3_O_4_ obtained using different concentrations of KMnO_4_ solution. Representative curves are shown from three independent experiments (n = 3).


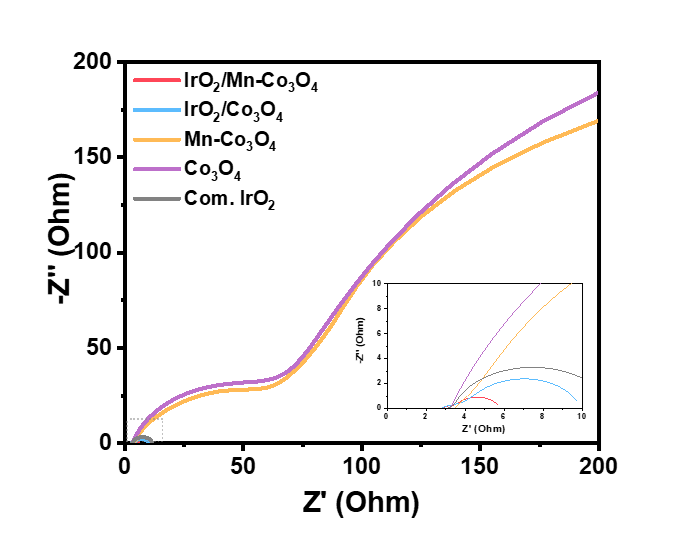


**Figure S12.** EIS of IrO_2_/Mn-Co_3_O_4_, IrO_2_/Co_3_O_4_, Mn-Co_3_O_4_, Co_3_O_4_ and Com. IrO_2_. Representative curves are shown from three independent experiments (n = 3)


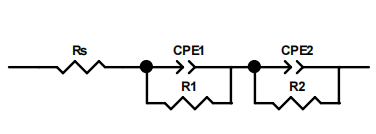


**Figure S13.** equivalent circuit model for EIS fitting

**Figure S14.** CV curves of (a) IrO_2_/Mn-Co_3_O_4_, (b) IrO_2_/Co_3_O_4_, (c) Mn-Co_3_O_4_, (d) Co_3_O_4_ and (e) Com. IrO_2_ at different scan rates. Representative curves are shown from three independent experiments (n = 3)

**
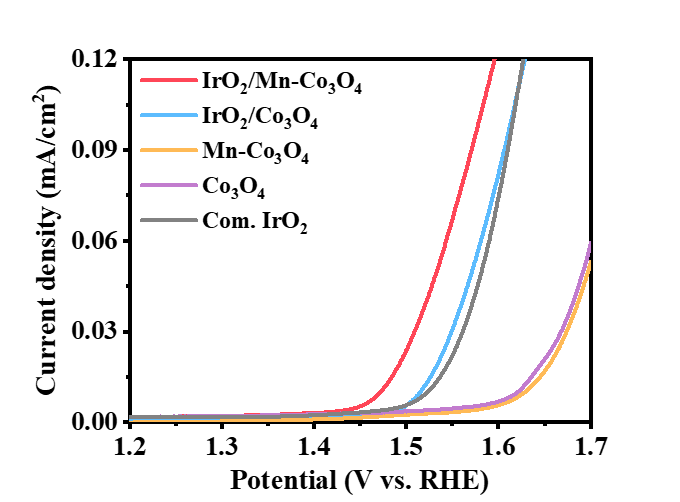
**

**Figure S15.** ECSA-normalized LSV curves of IrO_2_/Mn-Co_3_O_4_, IrO_2_/Co_3_O_4_, Mn-Co_3_O_4_, Co_3_O_4_ and Com. IrO_2_ in 0.5 M H_2_SO_4_. Representative curves are shown from three independent experiments (n = 3)


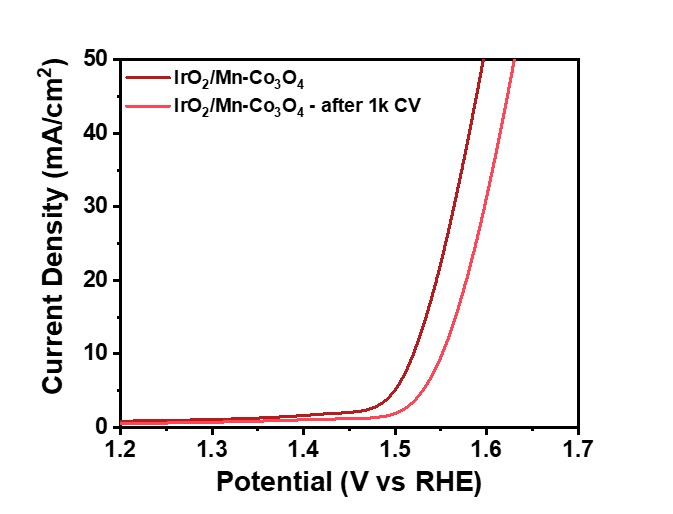


**Figure S16.** iR corrected LSV curves of IrO_2_/Mn-Co_3_O_4_ of before and after 1000 CV cycles. Representative curves are shown from three independent experiments (n = 3).

**Figure S17.** XRD patterns of IrO_2_/Mn-Co_3_O_4_ and IrO_2_/Co_3_O_4_ post-reaction (after CV test) states.


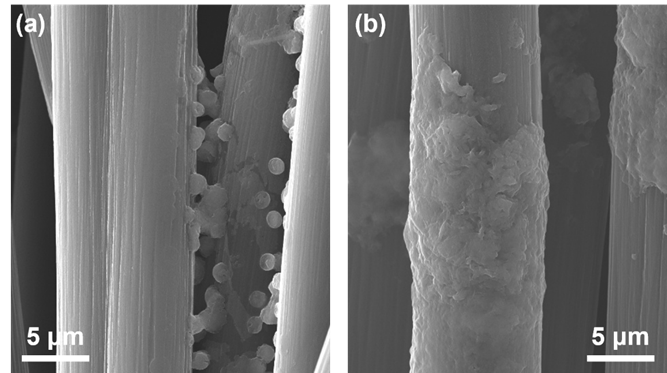


**Figure S18.** SEM images of (a) IrO_2_/Mn-Co_3_O_4_ after OER, (b) IrO_2_/Co_3_O_4_ after OER.


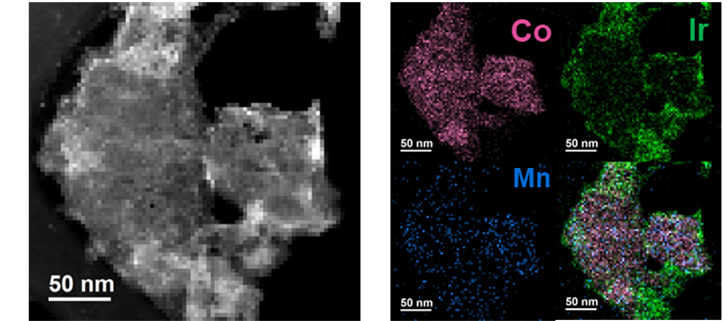


**Figure S19.** AC-HAADF-STEM and EDS mapping of IrO_2_/Mn-Co_3_O_4_ post-reaction.

**Figure S20.** Comparison of the calculated oxygen vacancy formation energies (E_v_) for pristine Co_3_O_4_ and Mn-Co_3_O_4_.


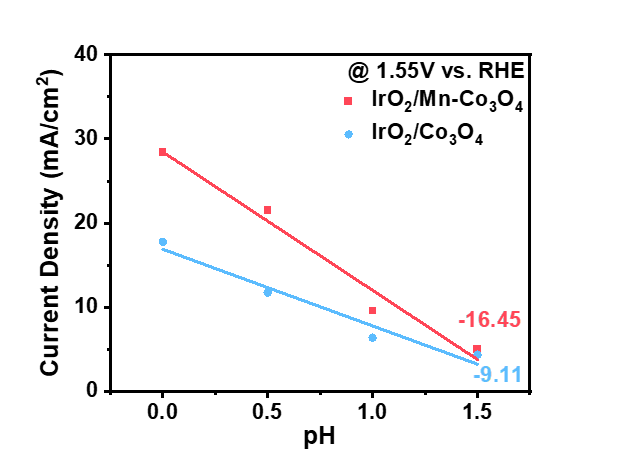


**Figure S21.** OER current density at 1.55 V RHE plotted in log scale as a function of pH, from which the proton reaction orders (ρH = ∂logj/∂pH) were calculated.


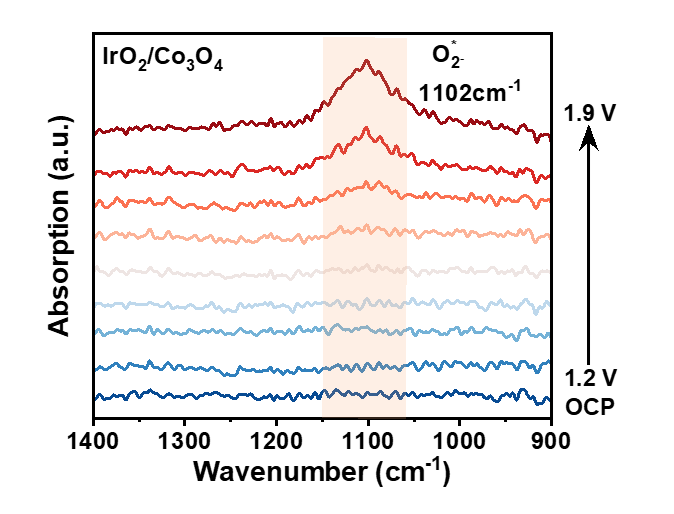


**Figure S22.** In-situ ATR-FTIR spectra results of IrO_2_/Co_3_O_4_.


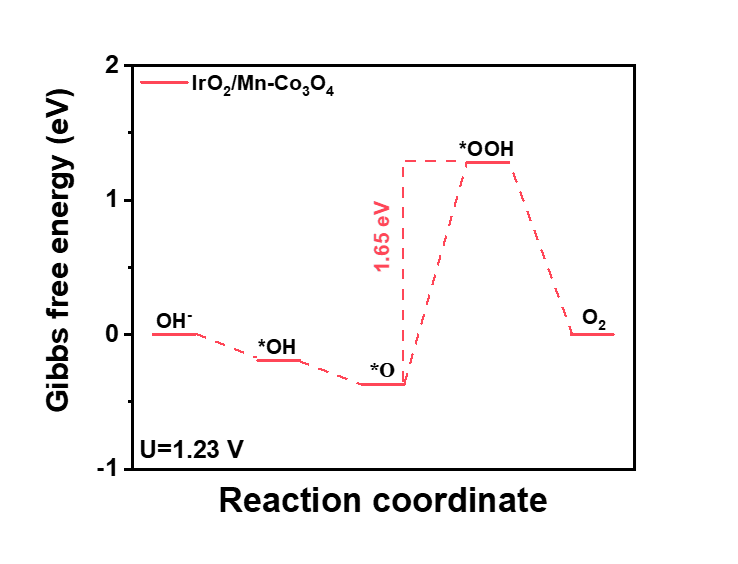


**Figure S23.** Gibbs free energy illustration by IrO_2_/Mn-Co_3_O_4_ catalysts during the OER process by AEM pathways.


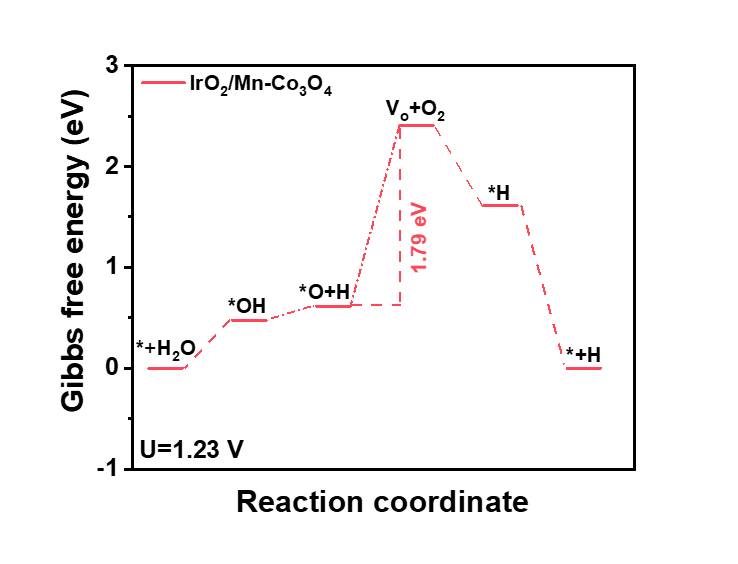


**Figure S24.** Gibbs free energy illustration by IrO_2_/Mn-Co_3_O_4_ catalysts during the OER process by LOM pathways.

**Figure S25.** Calculated Gibbs free adsorption energies of *OH (ΔG_*OH_) on the Co site and Ir site within the IrO_2_/Mn-Co_3_O_4_ catalyst model.

**Table S1.** ICP-OES data of IrO_2_/Mn-Co_3_O_4_ and IrO_2_/Co_3_O_4_

| **Catalysts** | **Mn**  **μg/mL** | **Co**  **μg/mL** | **Ir**  **μg/mL** |
| --- | --- | --- | --- |
| IrO_2_/Mn-Co_3_O_4_ | 0.55 | 11.5 | 2.77 |
| IrO_2_/ Co_3_O_4_ | / | 8.66 | 1.97 |

**Table S2.** Curve-fit Parameters a for Co K-edge EXAFS of IrO_2_/Mn-Co_3_O_4_ and IrO_2_/Co_3_O_4_ catalysts.

| **Catalysts** | Paths | *d* (Å) *^b^* | C. N. *^c^* | Δ*E*_0_ (eV) | *σ*^2^ (Å^2^) *^d^* | |
| --- | --- | --- | --- | --- | --- | --- |
| **IrO_2_/Co_3_O_4_** | Co-O | 1.93 ± 0.02 | 5.3 ± 1.3 | -1 ± 2 | | 0.01 |
|  | Co-Co | 2.89 ± 0.02 | 8.4 ± 4.1 |  |  | 0.02 |
| **IrO_2_/Mn-Co_3_O_4_** | Co-O | 1.93 ± 0.02 | 4.9 ± 1.1 | -1 ± 2 | | 1.01 |
|  | Co-Co | 2.88 ± 0.02 | 7.3 ± 3.7 |  |  | 2.42 |

*a*: The data ranges used in the fit are 3.0 ≤ *k* ≤ 11.0 Å^-1^ and 1.1 ≤ *R* ≤ 2.8 Å. *S_0_^2^* was fixed at 0.612, obtained from the Co foil measured at the same time. The number of variable parameters is out of independent data points. *R*-factors for these fittings are all below 0.02. *b*: The half path length. The paths for Co-O and Co-Co are from the crystal structure of Co_3_O_4_ (Fm-3m, COD 1526734) and CoO (Fm-3m, COD 1533087). *c*: average coordination number. *d*: Debye-Waller factor.

**Table S3.** Differential Charge Density of IrO_2_/Co_3_O_4_ and IrO_2_/Mn-Co_3_O_4_

|  | Ir | Mn | Co |
| --- | --- | --- | --- |
| IrO_2_/Mn-Co_3_O_4_ | 8.031 (-0.969) | 5.472(-1.528) | 7.743(-1.257) |
| IrO_2_/ Co_3_O_4_ | 8.018 (-0.982) | / | - 7.737(-1.263) |

**Table S4.** ICP-OES data of IrO_2_/Mn-Co_3_O_4_ and IrO_2_/Co_3_O_4_

| Element | Retention Rate (%) | Role and Observations |
| --- | --- | --- |
| Co | 55.8 % | Surface species inevitably undergo dissolution, while the bulk framework remains stable. |
| Mn | 85.3 % | Acts as a robust structural stabilizer; strongly anchored within the spinel lattice. |
| Ir | 82.7 % | Stabilized by strengthened Mn–O–Ir interfacial interactions. |

Retention (%) = (m_initial_ - m_dissolved_ ) / m_initial_ x 100%; **m_initial_**: Calculated from the total catalyst loading ( 0.750 mg cm ^-2^) and the weight percentages determined by ICP-OES (Ir: 14.5 %, Mn: 2.9 %, Co: 82.6 %); **m_dissolved_**: The cumulative mass of metal ions leached into the electrolyte as measured by ICP-OES after 100 h of operation.

**Table S5.** Comparison of the PEMWE performance of IrO_2_/Mn-Co_3_O_4_ with recently

reported catalysts.

| Catalyst | Feed | Temperature  (℃) | E_cell_ @1 A cm^-2^  (V) | Stability  (h) | Ref. |
| --- | --- | --- | --- | --- | --- |
| **IrO_2_/Mn-Co_3_O_4_** | **H_2_O** | **60** | **1.75** | **400@1000mA cm^-2^** | **This work** |
| Tm_0.1_Ir_0.8_O_2-δ_ | H_2_O | 50 | 1.766 | 500@1500 mA cm^-2^ | [5] |
| Ir/TiO_2_-MoO_x_ | H_2_O | 80 | 1.74 | 50@1000mA cm^-2^ | [6] |
| Ir/WO_x_ NSs | H_2_O | 80 | 1.71 | 500@1000 mA cm^-2^ | [7] |
| Ir_1_Ru/TiO_2_ | H_2_O | 80 | 1.611 | 200@2000 mA cm^-2^ | [8] |
| Ir/MnO_x_ | H_2_O | 80 | 1.63 | 300@1000 mA cm^-2^ | [9] |
| IrO_2_@TiO_2_ | H_2_O | 80 | 1.78 | / | [10] |
| IrO_2_@TaB_2_ | H_2_O | 80 | 1.83 | 100@1000 mA cm^-2^ | [11] |
| IrO_x_/Zr_2_ON_2_ | H_2_O | 80 | 1.927 | 60@1000 mA cm^-2^ | [12] |
| La-RuO_2_@TM | H_2_O | 60 | 1.815 | 120@1000 mA cm^-2^ | [13] |
| RuO_2_/MoO_3_-SO_4_ | H_2_O | 50 | 1.75 | 150@500 mA cm^-2^ | [14] |
| Ru/RuO_2_ | H_2_O | 50 | ~1.86 | 120@100 mA cm^-2^ | [15] |

**Table S6.** the d-band center of Ir on the Mn-modified surface

|  | Co-IrO_2_/Co_3_O_4_ | Co-IrO_2_/Mn-Co_3_O_4_ | Ir-IrO_2_/Co_3_O_4_ | Ir-IrO_2_/Mn-Co_3_O_4_ | Mn-IrO_2_/Mn-Co_3_O_4_ |
| --- | --- | --- | --- | --- | --- |
| up | -1.402 | -1.659 | -1.642 | -2.009 | 1.825 |
| down | -1.422 | -1.113 | -1.666 | -1.596 | -2.854 |
| average | -1.449 | -1.435 | -1.654 | -1.815 | -0.734 |

**Supplementary Reference**

1. G Kresse, Furthmüller J, "Efficiency of ab-initio total energy calculations for metals and semiconductors using a plane-wave basis set," *Computational Materials Science* 6 (1996): 15-50.

2. S Grimme, Antony J, Ehrlich S, et al., "A consistent and accurate ab initio parametrization of density functional dispersion correction (DFT-D) for the 94 elements H-Pu," *The Journal of Chemical Physics* 132 (2010):

3. V Wang, Xu N, Liu J-C, et al., "VASPKIT: A user-friendly interface facilitating high-throughput computing and analysis using VASP code," *Computer Physics Communications* 267 (2021): 108033.

4. S Maintz, Deringer VL, Tchougréeff AL, et al., "LOBSTER: A tool to extract chemical bonding from plane-wave based DFT," *Journal of Computational Chemistry* 37 (2016): 1030-1035.

5. S Hao, Sheng H, Liu M, et al., "Torsion strained iridium oxide for efficient acidic water oxidation in proton exchange membrane electrolyzers," *Nature Nanotechnology* 16 (2021): 1371-1377.

6. E-J Kim, Shin J, Bak J, et al., "Stabilizing role of Mo in TiO_2_-MoO_x_ supported Ir catalyst toward oxygen evolution reaction," *Applied Catalysis B: Environmental* 280 (2021): 119433.

7. J Cai, Huang H, Chen W, et al., "Two-dimensionally confined Ir/WO_x_ heterointerfaces boost the acidic oxygen evolution reaction for ampere-level stable PEM water electrolysis," *Science China Materials* 68 (2025): 2388-2396.

8. S Li, Deng L, Hung S-F, et al., "Embedded Ir─Ru Single-Atom Alloy with Self-Limiting Motifs for Sustainable Proton Exchange Membrane Water Electrolysis," *Advanced Materials* 38 (2025): e07340.

9. D Wang, Lin F, Luo H, et al., "Ir-O-Mn embedded in porous nanosheets enhances charge transfer in low-iridium PEM electrolyzers," *Nature Communications* 16 (2025): 181.

10. CV Pham, Bühler M, Knöppel J, et al., "IrO_2_ coated TiO_2_ core-shell microparticles advance performance of low loading proton exchange membrane water electrolyzers," *Applied Catalysis B: Environmental* 269 (2020): 118762.

11. Y Wang, Zhang M, Kang Z, et al., "Nano-metal diborides-supported anode catalyst with strongly coupled TaO_x_/IrO_2_ catalytic layer for low-iridium-loading proton exchange membrane electrolyzer," *Nature Communications* 14 (2023): 5119.

12. C Lee, Shin K, Park Y, et al., "Catalyst-Support Interactions in Zr_2_ON_2_-Supported IrO_x_ Electrocatalysts to Break the Trade-Off Relationship Between the Activity and Stability in the Acidic Oxygen Evolution Reaction," *Advanced Functional Materials* 33 (2023): 2301557.

13. X-Y Zhang, Yin H, Dang C-C, et al., "Unlocking Enhanced Catalysis Stability in Acidic Oxygen Evolution: Structural Insights for PEM Applications under High-Current Density," *Angewandte Chemie International Edition* 64 (2025): e202425569.

14. Y Duan, Wang L-L, Zheng W-X, et al., "Oxyanion Engineering on RuO_2_ for Efficient Proton Exchange Membrane Water Electrolysis," *Angewandte Chemie International Edition* 63 (2024): e202413653.

15. Q Lu, Liu J, Zou X, et al., "Breaking the Activity-Stability Trade-Off of RuO_2_ via Metallic Ru Bilateral Regulation for Acidic Oxygen Evolution Reaction," *Angewandte Chemie International Edition* 64 (2025): e202503733.
